# Avoiding Biased-Feeding in the Scheduling of Collaborative Multipath TCP

**DOI:** 10.1371/journal.pone.0161213

**Published:** 2016-08-16

**Authors:** Meng-Hsun Tsai, Chien-Ming Chou, Kun-chan Lan

**Affiliations:** Department of Computer Science and Information Engineering, National Cheng Kung University, Tainan, Taiwan; University of Texas at San Antonio, UNITED STATES

## Abstract

Smartphones have become the major communication and portable computing devices that access the Internet through Wi-Fi or mobile networks. Unfortunately, users without a mobile data subscription can only access the Internet at limited locations, such as hotspots. In this paper, we propose a collaborative bandwidth sharing protocol (CBSP) built on top of MultiPath TCP (MPTCP). CBSP enables users to buy bandwidth on demand from neighbors (called Helpers) and uses virtual interfaces to bind the subflows of MPTCP to avoid modifying the implementation of MPTCP. However, although MPTCP provides the required multi-homing functionality for bandwidth sharing, the current packet scheduling in collaborative MPTCP (e.g., Co-MPTCP) leads to the so-called biased-feeding problem. In this problem, the fastest link might always be selected to send packets whenever it has available cwnd, which results in other links not being fully utilized. In this work, we set out to design an algorithm, called Scheduled Window-based Transmission Control (SWTC), to improve the performance of packet scheduling in MPTCP, and we perform extensive simulations to evaluate its performance.

## Introduction

There are now more than 1.7 billion smartphones in the world, and the penetration rate is close to 25% [1]. Smartphones have thus become major communication and portable computing devices in recent years. However, in most cities, Wi-Fi hotspots are intermittent, with limited radio transmission range [2]. Although 3G and Long Term Evolution (LTE) provide excellent coverage, not everyone is willing to pay for a 3G/4G subscription (the penetration rate of global mobile broadband connections was still under 40% by the end of 2014 [3]). In other words, many smartphone users often have no mobile Internet connection. To allow these users to access the Internet, one possible solution is to ‘buy’ unused bandwidth from neighboring smartphones that have Internet connections [4].

Among all mobile Internet applications, video applications are the most popular (exceeding 55% of the total data consumption [5]). Video streaming is known to be bandwidth-hungry (e.g., 1080P High Definition (HD) video, 4K video, and panoramic video for interactive virtual cameras [6]), such that 3G bandwidth might be insufficient under certain circumstances (e.g., when radio signal quality is poor). Although 4G technologies, such as LTE, can provide more bandwidth, their penetration rate is currently rather low [3]. To obtain more bandwidth, various prior studies [4, 7, 8] have proposed the idea of bandwidth aggregation through multi-homing. For example, PRISM [7] employs an inverse multiplexer at the proxy server and implements a sender-side congestion control mechanism to aggregate bandwidth from neighboring nodes over TCP. In CStream [4], the video server distributes data frames via TCP connections over multiple Helpers. However, PRISM and CStream are both server-based solutions, which in practice are difficult to implement on every Internet server. Alternately, Co-MPTCP [8] extends the MultiPath TCP (MPTCP) at the receiver side to create a collaborative network. Nevertheless, Co-MPTCP does not address the issue of maintaining the multi-homed collaborative network. Furthermore, current implementations of packet scheduling in Co-MPTCP are based on round trip time (RTT), and they might result in the so-called biased-feeding [9, 10] problem and introduce many out-of-order packets, which significantly reduces the aggregated bandwidth.

In this paper, we propose a collaborative bandwidth sharing protocol (CBSP) to group neighboring smartphones and aggregate their unused bandwidth based on MPTCP. Our current implementation of CBSP focuses on TCP traffic because this is the dominant form (and HTTP streaming over TCP is the major bearer of mobile video traffic; specifically, 98% of multimedia streaming traffic in mobile traffic is delivered over HTTP [11]). CBSP is built on top of MPTCP standardized by IETF [12], and hundreds of millions of iPhones and iPads already support MPTCP. CBSP can also work together with various existing systems, such Webus [13] and on-bus Wi-Fi [14].

In CBSP, a virtual interface is created on the user’s smartphone for each neighboring smartphone (called a Helper) and bound to a subflow of MPTCP. CBSP uses a proxy server (called a CBSP server) as a middlebox to coordinate MPTCP connections. The user can access all Internet servers through the CBSP server, and so the Internet servers do not need to support MPTCP. In other words, CBSP protocol module only needs to be installed on the user’s smartphone. The CBSP server monitors all traffic forwarded by Helpers and maintains the accounting information (including pricing of the data, if necessary) of the users and Helpers. We propose a Scheduled Window-based Transmission Control (SWTC) scheduling algorithm to improve the performance of packet scheduling over MPTCP. The contributions of this work are twofold. (1) We propose a novel protocol, called CBSP, on top of MPTCP to allow smartphone users to utilize their neighbors’ unused bandwidth without the need to modify the Internet servers (i.e., to make them MPTCP-capable). (2) We propose a scheduling algorithm called SWTC to improve the performance of MPTCP.

The remainder of this paper is organized as follows. In Section 2, we survey the related studies. An overview of MPTCP and a discussion of CBSP are presented in Section 3 and Section 4, respectively. The performance of SWTC scheduling is evaluated and various simulation results are shown in Section 5. We discuss implementation details in Section 6. Finally, we conclude this paper and describe various future work in Section 7.

## Related Work

Our work is built on various previous studies of bandwidth sharing, bandwidth aggregation, and multi-homing packet scheduling, and these are outlined below.

### Bandwidth Sharing

There is a large body of work [15] in the context of mesh networks discussing how to share Internet connectivity among a group of nodes. In mesh networks, nodes in a neighborhood connect wirelessly to form a grid and share Internet access from one or a few nodes. Many of these studies focus on sharing a single connection between multiple devices, especially when this connection is temporarily idle, such as DOMS [16] and FatVAP [17]. DOMS constructs a number of “virtual” access points (APs), one for each of the surrounding Wi-Fi devices. A FatVAP client connects to multiple APs by using fast switches in kernel space. However, each client requires a modified driver to support FatVAP. All of these studies select a higher-bandwidth path to send packets. However, these earlier works did not consider the use of a bandwidth aggregation mechanism for bandwidth sharing, nor did they discuss how to efficiently schedule packets over multiple links.

### Bandwidth Aggregation

PRISM [7], CStream [4], and Co-MPTCP [8] aggregate bandwidth from neighboring nodes over TCP traffic. PRISM consists of an inverse multiplexer at the proxy (PRISM-IMUX) and a sender-side congestion control mechanism (TCP-PRISM) to achieve bandwidth aggregation. PRISM-IMUX captures each data packet transmitted between the sender and the receiver. TCP-PRISM selects a link with the minimum link utilization and the smallest expected arrival time (i.e., half RTT) to send a packet. However, PRISM needs to modify the TCP implementation on the sender side, which could hinder its deployment in the real world. CStream focuses on video streaming over TCP through aggregation of bandwidth from neighboring Helpers’ 3G connectivity. However, the related research did not discuss how to efficiently schedule packets over multiple links. CStream is a server-based approach, so it also has similar deployment issues to those seen with PRISM. Co-MPTCP extends the MPTCP at the receiver side and uses multiple ‘relay’ nodes to forward traffic to a ‘root’ node, but it does not address the biased-feeding problem [9, 10].

All these works assume that the Helpers are static nodes, so they do not address how to maintain these Helpers. In contrast, the current work takes the mobility of Helpers into consideration when selecting Helpers and scheduling packets.

How packets are scheduled over multiple paths can significantly affect the network performance in a multi-homed environment. The network metric used by the prior work to select a path has mostly been based on either estimated bandwidth or RTT.

### Multi-homing Packet Scheduling

EAPC [10] favors selecting non-congested links to send packets by estimating the available bandwidth and considering the congestion status of links. If all subflows are not congestion, the subflow is slected with the shortest smoothed RTT. EAPC is not adaptive, though, because the thresholds for detecting the congestion need to be predefined for different scenarios. In addition, in some cases, the congested links can be part of a faster path to the destination. One major problem with only considering the available bandwidth for packet scheduling is that it might be difficult to quickly and accurately reflect the network dynamics in a mobile scenario [18].

ODS [19] ranks its set of destination addresses using an estimated time of acknowledgement (ETA) by considering RTT with the estimated queuing delay of the bottleneck, finding the destination address with the minimum ETA, and then choosing the packet that is destined to that address and has the lowest sequence number to send. CWA-MPTCP [20] estimates the packet arrival time by RTT, using a delay ratio to indicate a high-delay path. However, this method must modify the congestion control algorithm of TCP. None of the above studies consider the lossy nature of wireless links when scheduling packets. Alternately, Fountain code-based Multipath TCP (FMTCP) [21] estimates packet arrival time by RTT and RTO with the loss rate. Finally, Co-MPTCP extends MPTCP (i.e., also based on RTT scheduling) and has a similar architecture to our CBSP protocol. It has been shown [10] that these prior extensions of MPTCP (which schedules packets based on RTT and/or the cwnd window) all suffer from the biased-feeding problem [10], in which, most of the time, only the fastest link is selected to send data, while all the other links are not utilized. In this paper, we propose a scheduling algorithm called SWTC, which considers the following metrics for selecting the path to send packets: expected delay using RTT with the loss rate, estimated available bandwidth, and estimated TCP cwnd. Table 1 shows a comparison of different packet scheduling algorithms and the network metrics used to select the path for sending a packet.

**Table 1:** Comparison of Different Packet Scheduling Algorithms.

Network metrics used	RTT	Estimated bandwidth	cwnd value	Loss rate
**SWTC**	✓	✓	✓	✓
**EAPC** [10]	✓	✓		
**ODS** [19]	✓			
**FMTCP** [21]	✓			✓
**CWA-MPTCP** [20]	✓		✓	
**Co-MPTCP** [8]	✓			

## An Overview of MPTCP

MPTCP has been standardized by IETF in RFC 6824 [12] to allow a TCP connection to use multiple paths to maximize resource usage and increase reliability. MPTCP can thus take one input data stream from an application and split it into one or more subflows by simultaneously using several interfaces (when available).

### Connection Setup and Shutdown

All control messages of MPTCP are carried by a TCP option field, such as MP_CAPABLE, MP_JOIN, and Data Sequence Signal (DSS). An MPTCP connection is set up by including an option MP_CAPABLE in the TCP headers in the three-way handshake. Specifically, the client sends a TCP segment with SYN/MP_CAPABLE to the server. If the server supports MPTCP, it responds to the TCP segment with SYN/ACK/MP_CAPABLE. The client responds to the TCP segment by sending ACK/MP_CAPABLE to the server to finish the connection setup. If the client wants to add more paths, it will start another round of the three-way handshake with the server for a new subflow by using MP_JOIN. When the client wants to inform the server that it has no more data to send, DATA_FIN is sent to close the MPTCP connection (i.e., all previously created subflows). Moreover, the client can also close only a specific subflow by RST without affecting the remaining subflows.

### Scheduling

The packet scheduler in MPTCP breaks the byte stream received from the application into segments to be sent on available subflows. MPTCP uses a 64-bit connection-level sequence number (called DSN) to number all data sent over the MPTCP connections and a 32-bit sequence number for the subflow sequence space (called DSS). The segments sent on different subflows are correctly re-ordered at the receiver through the 64-bit DSN. In addition, MPTCP uses a connection-level receiver buffer (we refer to it as a “reordering buffer”), into which segments are placed until they are in order and can be read by the application.

**Fig 1:**
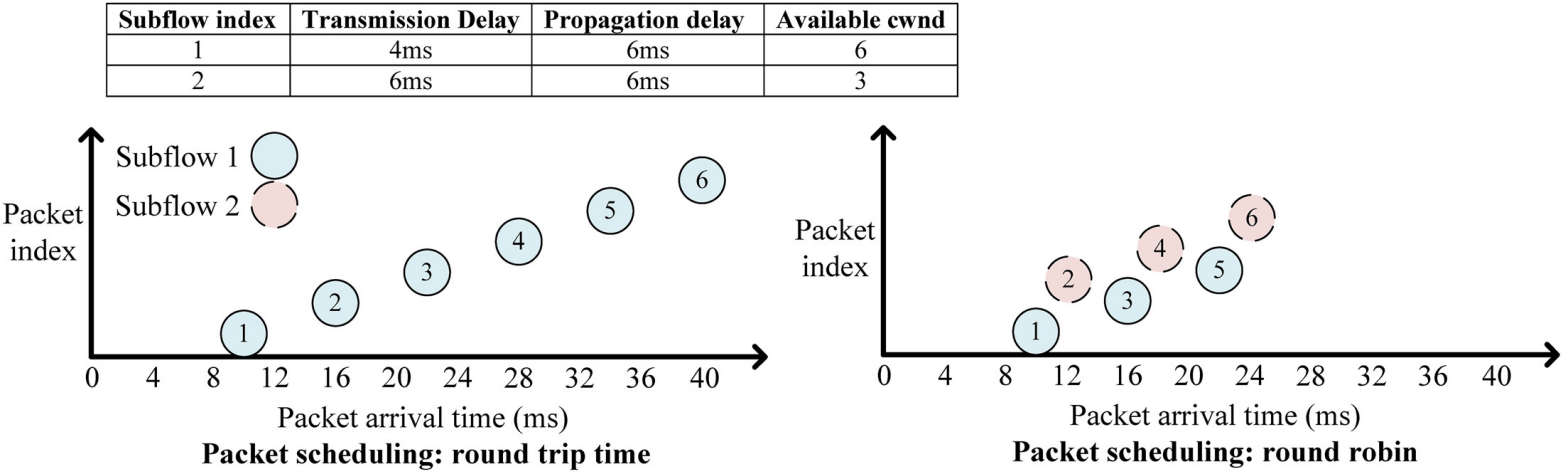
An example of biased feeding.

In MPTCP, when several subflows are available simultaneously, packets are scheduled to these according to the order of time-distance (i.e., RTT) [22]. In other words, MPTCP favors fast subflows. In addition, as in regular TCP, each subflow’s TCP’s congestion window (cwnd) needs to be checked before packets can be scheduled to that subflow [23]. Current implementations of packet scheduling in MPTCP [22, 24] may lead to the biased-feeding problem, in which the fastest link is always selected to send traffic and the other links are not utilized until the fastest link has no available cwnd, which could seriously decrease the parallelism of data transfer. As shown in the example in Fig 1, it could take 40 ms to transmit all the packets using RTT scheduling, while only 26 ms are required when a simple round-robin scheduling is employed.

### Error Control

MPTCP uses connection-level ACK (i.e., data sequence mapping) to provide a robust service to the application and decide when a connection-level segment is received, and data sequence mapping is part of the DSS option to provide a mapping from the subflow sequence number to the DSN. To maintain TCP semantics and trigger subflow-level retransmissions, subflow-level ACK is implemented as the regular TCP ACK.

When a segment is lost, the receiver detects the gap in the subflow-level ACK, and regular TCP retransmission mechanisms are triggered to recover from the loss, including fast retransmission, fast recovery, and timeout retransmission. The sender retransmits the lost data on the original subflow. In the meantime, the sender can also resend the lost data with the same DSN on a different subflow because the MPTCP specification does not mandate any mechanisms for handling retransmissions. Moreover, when a subflow fails, the sender can resend the lost data on the other available subflows and also continue trying to retransmit the lost data on the failed subflow.

## Collaborative Bandwidth Sharing Protocol (CBSP)

**Fig 2:**
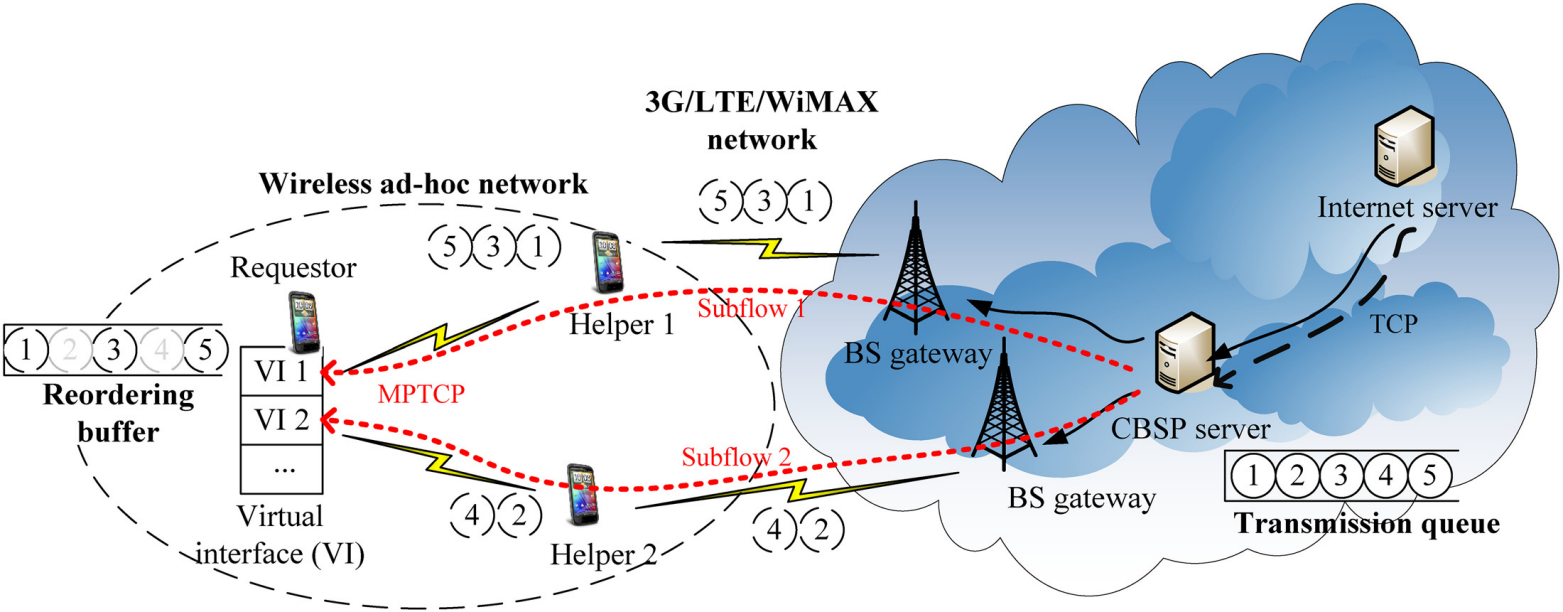
Bandwidth sharing over the MPTCP.

To allow a mobile device (e.g., a smartphone) to utilize the unused bandwidth of neighboring nodes, we propose a bandwidth sharing technique named CBSP that is built on top of Multipath TCP (MPTCP). As shown in Fig 2, our architecture consists of mobile nodes (i.e., smartphone users) and a CBSP server. We consider two types of nodes, Requestors and Helpers, which run the CBSP client program and together can form an ad-hoc network using their local wireless network connectivity (e.g., Wi-Fi), while the CBSP server is located somewhere on the Internet (here, we assume the IP address of the CBSP server is fixed and known by all CBSP clients).

The CBSP server can be the accounting server for counting the packets forwarded by each Helper (e.g., through monitoring the headers of the tunneled IP-in-IP packets and NAT port numbers). When a requestor wants to buy bandwidth from its neighbors (here, we assume that Helpers are selfish, and thus the requestor might need to ‘buy’ bandwidth from Helpers through some virtual credit mechanisms [25]), it first broadcasts an Invite message to all neighboring nodes and then forms an ad-hoc network with those that send back a Join message (i.e., the potential Helpers). The requestor treats these Helpers as its ‘virtual interfaces (VI)’ and strips its traffic over these on top of MPTCP toward the CBSP server. Once packets from multiple Helpers are in order, the CBSP server then forwards them to the destination (e.g., an Internet server). Similarly, in the case of downlink traffic, a connection from the Internet server is packet-stripped at the CBSP server and then sent to different Helpers via MPTCP. When a Helper receives the data from the CBSP server, the packets are forwarded toward the requestor via Wi-Fi. Packets from multiple Helpers are reassembled at the requestor and forwarded to the corresponding application once they are in-order. In addition, we employ the Scheduled Window-based Transmission Control (SWTC) scheduling algorithm (the details of which are discussed later) to improve the performance of MPTCP in CBSP.

### Protocol Data Flow

**Fig 3:**
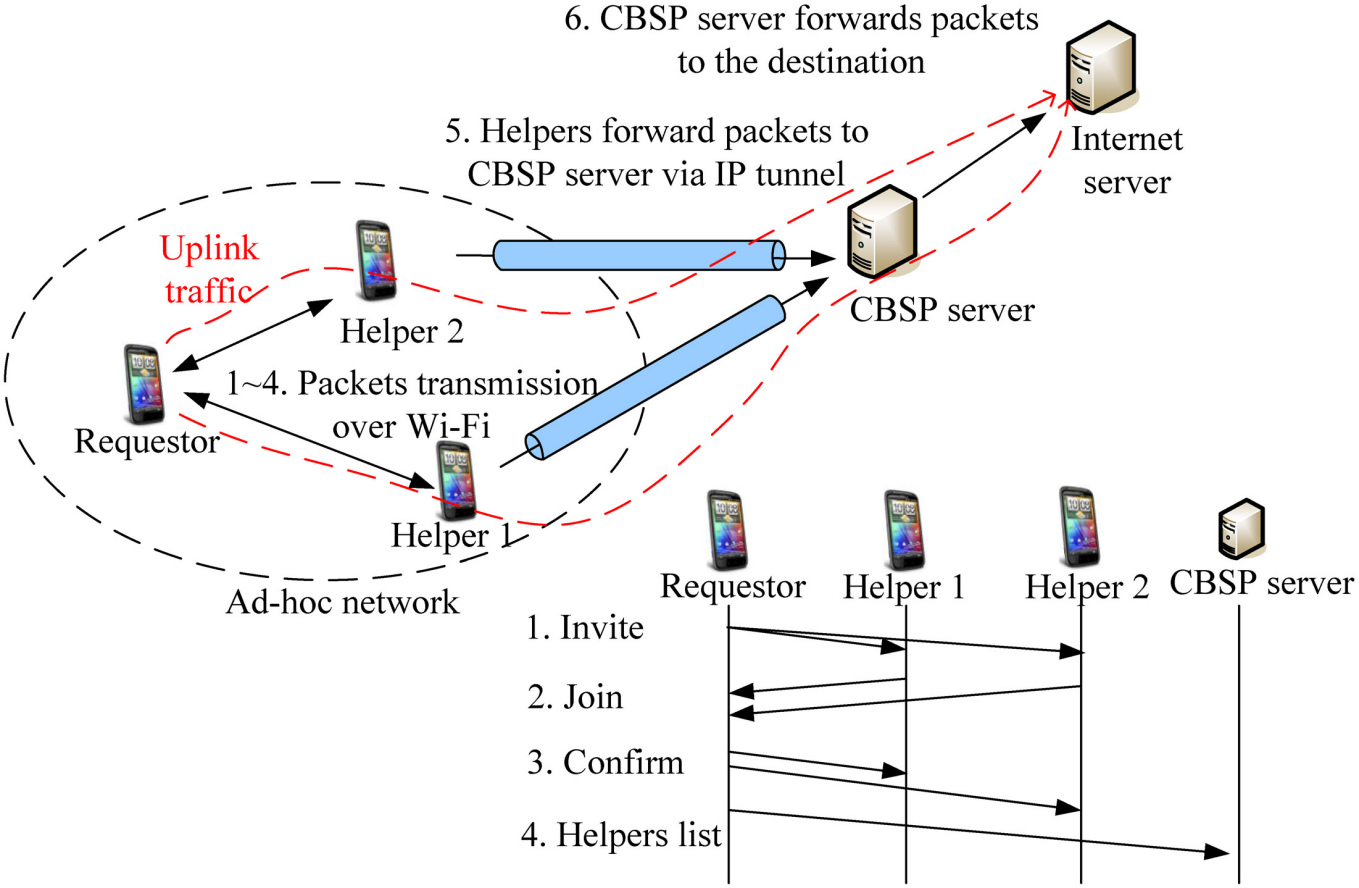
The protocol data flow.

The data flow of the CBSP protocol is shown in Fig 3. The requestor node first broadcasts Invite messages to its neighboring nodes. Some neighbors then reply back with Join messages if they are willing to sell their unused bandwidth. Next, a Confirm message is sent to inform the selected neighbors (i.e., the Helpers) that an ad-hoc network has been created (e.g., over Wi-Fi) between them and the requestor, and a Helper list is also sent to the CBSP server from the requestor. Once the ad-hoc network is in place, the requestor strips its uplink traffic across multiple Helpers and tunnels them over MPTCP to the CBSP server. Finally, the CBSP server forwards all the received packets to the destination (e.g., the Internet server) once they are in order. In the meantime, each Helper could generate a bill for the packets it forwards for the requestor and sends to the CBSP server, which then creates a receipt for each (requestor, Helper) pair.

### Protocol Functions

**Fig 4:**
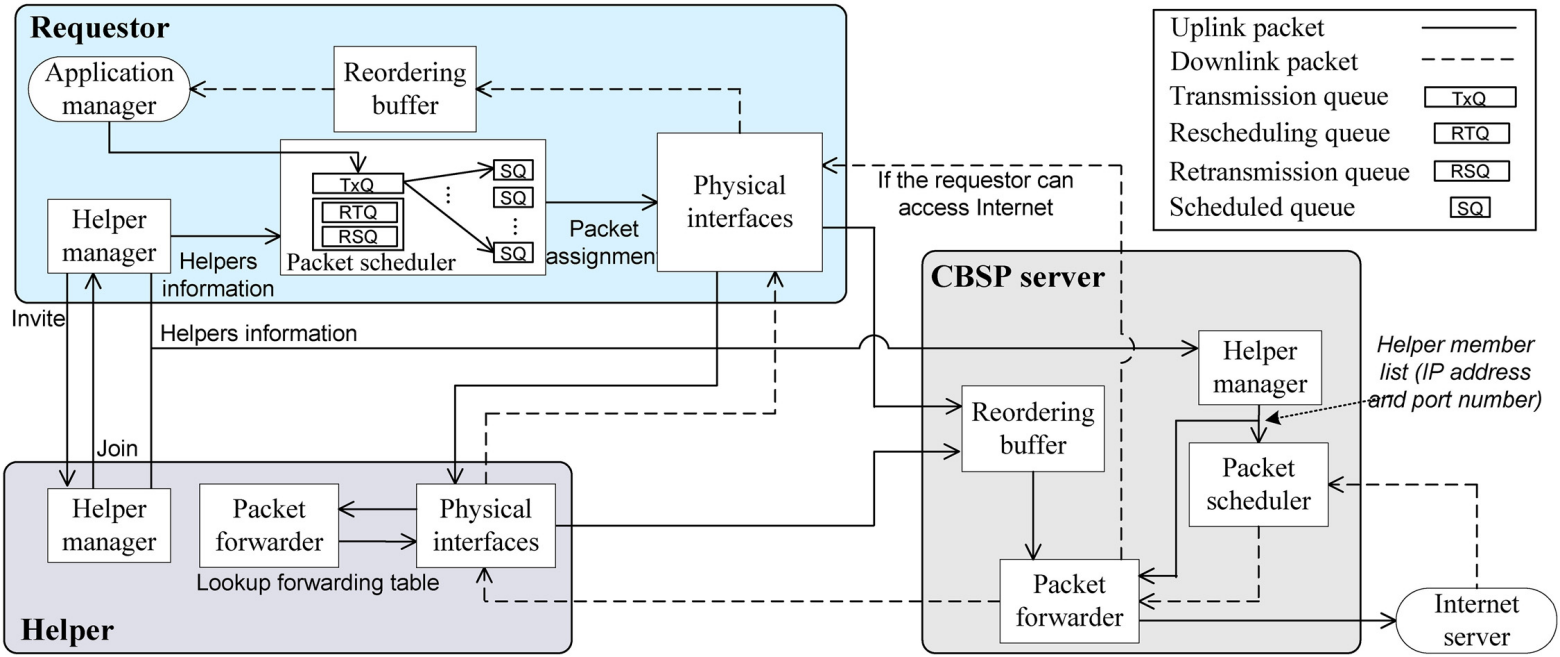
The functional blocks of CBSP.

The functional blocks of CBSP are shown in Fig 4. There are three types of nodes: Requestor, Helper, and CBSP server. In the requestor node, the application manager assigns a port number to each application that runs on top of the CBSP protocol, while the Helper manager in the Requestor node is responsible for Helper discovery and creation of an ad-hoc network between the requestor and Helpers. In addition, the Helper manager also maintains a Helper list that contains all information of selected Helpers and the path characteristics between each Helper and the CBSP server. The Helper information is used by the packet scheduler to decide how to schedule packets through multiple Helpers. In the packet scheduler, the traffic is first put into the transmission queue (TxQ) and then assigned to different scheduled queues (SQ) based on the results of SWTC scheduling. One SQ is created for each Helper and is regarded as a virtual interface (VI) of the requestor. One additional SQ is created for the requestor if it also has a physical network interface (e.g., 3G or LTE) to connect to the Internet. In Fig 4, RTQ is the retransmission queue, which stores the packets already sent out but not yet received by their ACKs. RSQ is the rescheduling queue. CBSP uses RSQ to manage the case in which some Helpers leave the network in the middle of traffic transmission, which will be discussed in later sections.

**Fig 5:**
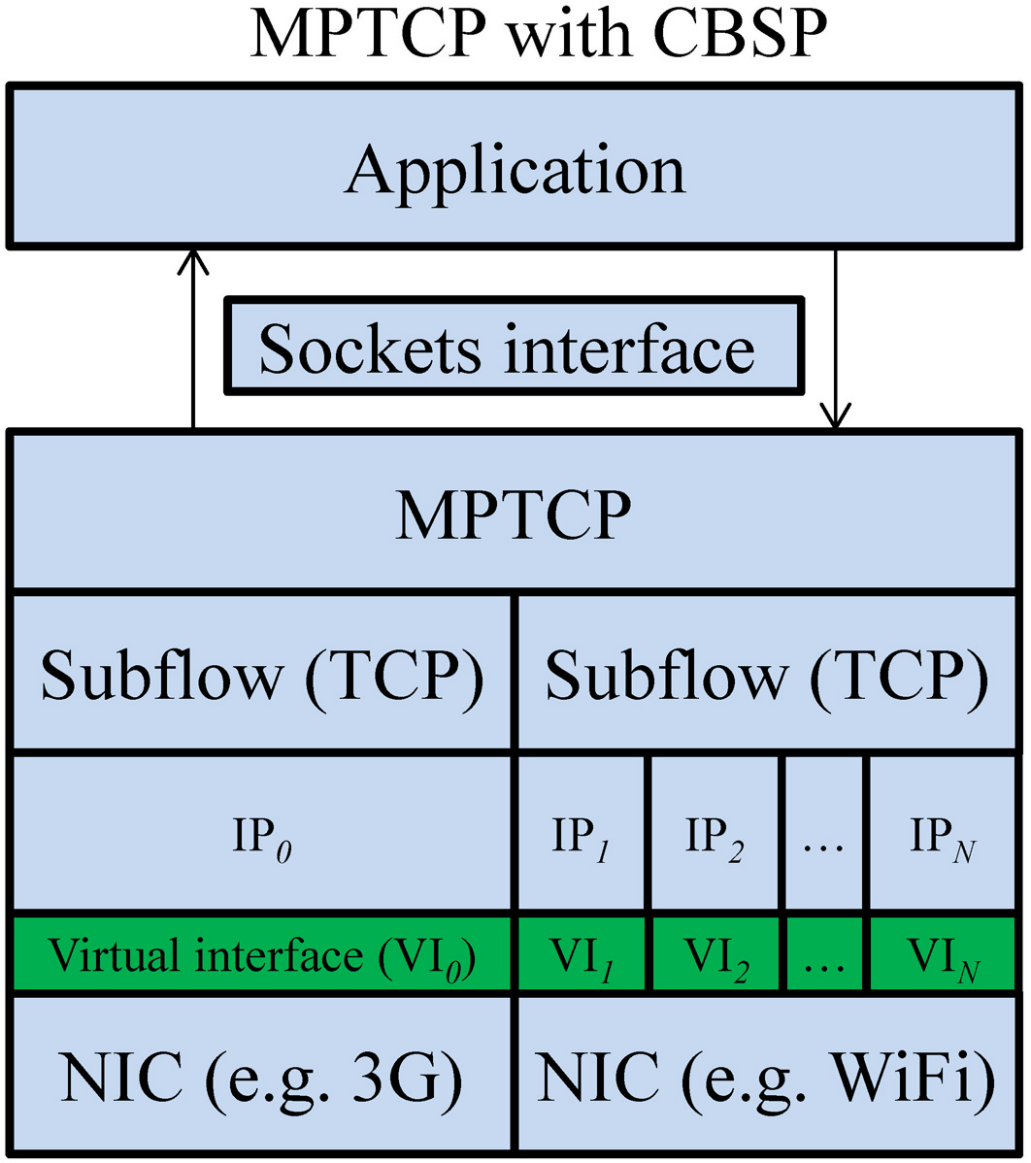
The protocol stack of MPTCP with CBSP.

When a new Helper joins the network, a new virtual interface will be created for this Helper, as shown in Fig 5. In the case that the requestor has its own wide-area network (WAN) interface (e.g., 3G) to the Internet, this interface will then be considered as virtual interface 0 (*VI*_*0*_). The mapping between virtual interfaces (*VI*_*1*_, …, *VI*_*n*_) and the corresponding Helper is handled by the packet scheduler. In addition, the requestor should periodically update the “Helper information” (e.g., the list of Helpers and their IP addresses and port numbers) with the CBSP server. Packets received at the requestor or the CBSP server are first stored in the reordering buffer until they are in order and are then forwarded by the application manager to the corresponding application or by the packet forwarder to the destination (e.g., an Internet server). From the user’s viewpoint, the virtual interface is an extra network interface. Based on the Helper and path information obtained from the Helper manager, the packet scheduler then strips packets over multiple virtual interfaces through the physical wireless interfaces (e.g., Wi-Fi).

### Connection Setup and Data Flow of the Protocol

**Fig 6:**
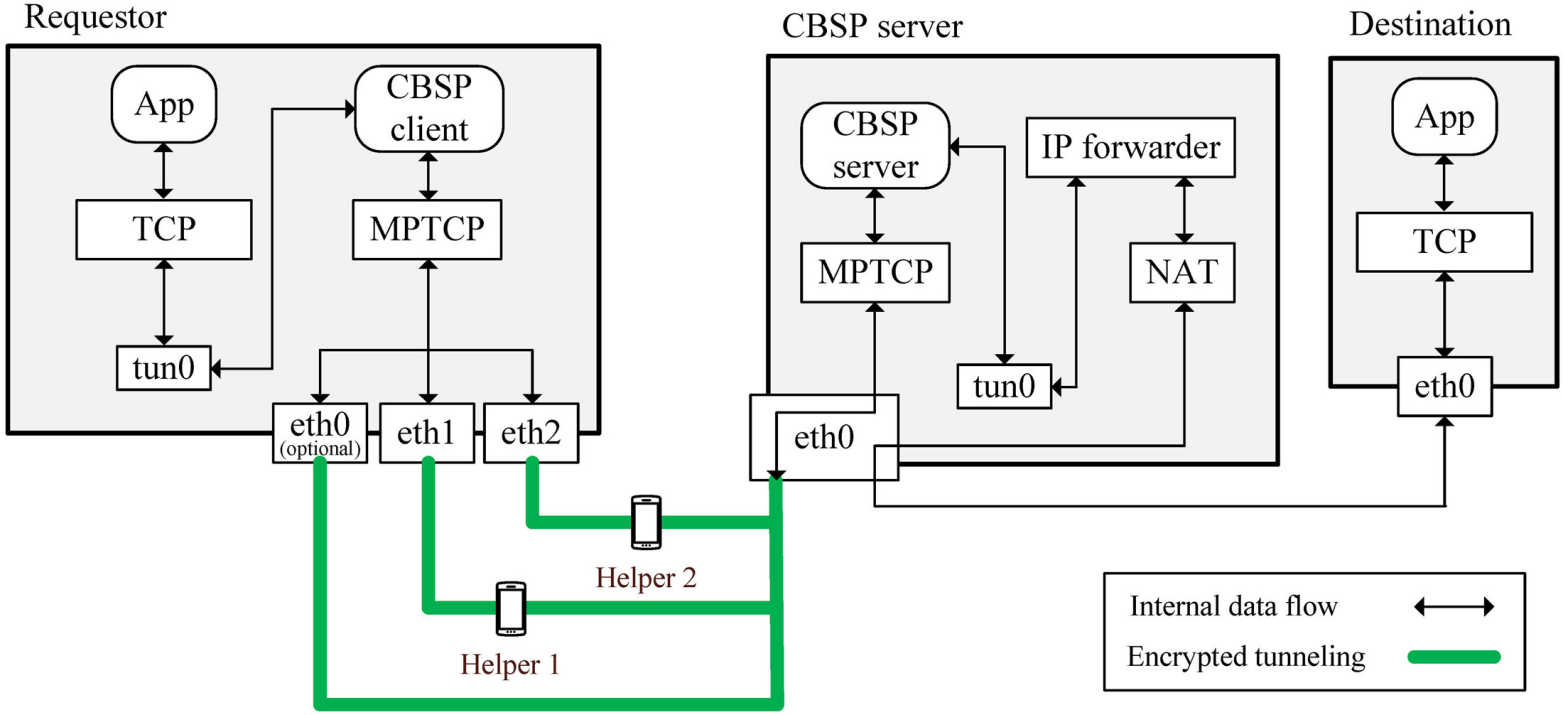
The data flow of CBSP.

The Requestor and the Helper nodes run the CBSP client program. When the client nodes run CBSP for the first time, they need to register with the CBSP server, which acts as a Certificate Authority (CA) for the CBSP service. When a requestor has some data to send, it sets the program to the ‘Request mode,’ which enables the program to periodically broadcast ‘Invite’ messages until some Helpers are found. Alternately, when the CBSP client program is in ‘Help mode,’ the node becomes a Helper, which listens to ‘Invite’ messages and responds with a ‘Join’ message (the requestor and the Helper nodes also use a public-key mechanism to authenticate each other’s Invite/Join messages). After receiving the ‘Join’ message, the requestor replies with a ‘Confirm’ message and assigns an IP address to each Helper. After the Helper receives the ‘Confirm’ message, it starts forwarding all the traffic from the requestor to the CBSP server. Once the requestor can connect to the CBSP server, it sets up an MPTCP connection with the CBSP server through its Helpers. All packets traveling between the requestor and the CBSP server are tunneled and encrypted with a PKI-like mechanism to ensure security, as shown in Fig 6. More specifically, a tunnel (e.g., tun0 in Fig 6) is created at the requestor, and the routing table is modified so that all application traffic is directed to tun0 as the default route. The CBSP client continuously reads packets from tun0 and sends them over MPTCP to the CBSP server via its virtual interfaces. The CBSP server enables IP forwarding and network address translation (NAT) to forward the requestor’s traffic (the Helper also needs to enable IP forwarding), and thus the requestor can access the Internet through the CBSP server. The requestor can add more Helpers (i.e., subflows) when needed through the MP_ADDR option of the MPTCP packet.

### Helper Selection

To recruit a Helper, the requestor first broadcasts the Invite message, which contains the IP address of the CBSP server, the wireless network information, such as the network service set identification (SSID), and so on. Upon receiving the Invite message, a neighboring node sends back a Join message if it is willing to sell its bandwidth. The Join message contains the packet price, the remaining energy of the responding neighbor, and the path characteristics [26] between the CBSP server and this potential Helper, such as the RTT, available bandwidth, loss rate, and node mobility information if available (e.g., through GPS). The requestor broadcasts an Invite message every *T* seconds when its bandwidth need is not met. The timer period *T* will be increased exponentially [27] until the requestor has finally received enough Join messages (i.e., the offered aggregated bandwidth can satisfy the requestor’s bandwidth needs) from its neighboring nodes. The Helper selection does not start until this procedure has been run for some time (which can be user-defined). When selecting the Helpers, the requestor will generally prefer the ones that charge a lower packet price and will use their mobility and network throughput information as a tie-breaker.

When mobility information is available, the requestor can estimate the encounter duration *δ*_*i*_ of Helper *i* as follows [28].

$$\delta_{i}=\frac{-\left(d_{x}R_{x}^{}+d_{y}R_{y}^{}\right)+\sqrt{\left(R_{x}^{2}+R_{y}^{2}\right)r^{2}-{\left(d_{y}R_{x}^{}-d_{x}R_{y}^{}\right)}^{2}}}{R_{x}^{2}+R_{y}^{2}}$$ (1)

where *R*_*x*_ and *R*_*y*_ are the relative velocities between two nodes in the *x* and *y* directions, *d*_*x*_ and *d*_*y*_ are the distances between two nodes in the *x* and *y* directions, and *r* is the transmission range.

In addition, because smartphones have limited battery lifetime, we need to consider the battery lifetime of the Helper. The battery lifetime (*β*_*i*_) of Helper *i* can be estimated by *β*_*i*_ = *remaining power/the rate of consumption*, in which the rate of consumption can be predicted, as discussed in an earlier work [29].

Using the estimated encounter duration and battery lifetime information, the requestor can then calculate the available encounter duration (denoted as *ε*_*i*_), which is the period during which the Helpers are within the radio range of the requestor and still have power. That is, *ε*_*i*_ = min(*β*_*i*_, *δ*_*i*_).

To record the historical throughput of each Helper, the CBSP server maintains a bandwidth map [30] that is collected by the CBSP client nodes. The CBSP clients passively measure the path characteristics [26] between them and the CBSP server periodically and update the database in the CBSP server (note that such information can be piggybacked to the CBSP server through normal data traffic).

When the requestor does not have its own WAN interface, the first neighboring node that responds to the Invite message will always be selected as one of the Helpers. The requestor can then use this Helper to access the database in the CBSP server to select the remaining Helpers. Initially, there might be no historical data in the CBSP server’s database. In that case, we can compare the throughput of different Helpers based on their provided network type (e.g., HSDPA > UMTS > EDGE > GPRS) or signal strength levels.

The requestor generally selects the Helpers that charge a lower packet price for forwarding packets (although this might achieve sub-optimal performance, which will be discussed in later sections). If the prices demanded by potential Helpers are the same, the requestor selects the Helpers based on the available encounter duration (*ε*_*i*_) and the data throughput (*S*_*i*_) of its neighboring nodes. The amount of data *A*_*i*_ that Helper *i* can deliver during its encounter with the requestor can be estimated as *A*_*i*_ = *S*_*i*_ ×*ε*_*i*_. The requestor picks the first *h* Helpers who have the highest *A*_*i*_ with an aggregate bandwidth (*σ*) greater than the minimum bandwidth requirement (*ρ*) of the requestor. In other words, $\sigma =\sum_{i=1}^{h}S_{i},\;\sigma \geq \rho$, where *S*_*i*_ is the throughput that can be provided by Helper *i*.

When *σ* falls below *ρ* (e.g., when some Helpers leave), the requestor restarts the Helper discovery procedure. Note that packet scheduling in CBSP is independent of the neighbor selection process. The packet scheduler can still schedule packets over the selected Helpers regardless of whether the requirement *σ* is met or not.

To detect if some Helper has left the network, we employ the following approach: if the requestor has not received/overheard any packet from a Helper for a given period of time, it will send out a Hello packet to this Helper. In the meantime, the packet scheduler will not schedule any packets to this Helper until the requestor receives a Hello-ACK back from it. Each Hello packet is associated with a timer. The requestor tries to probe this Helper a given number of times (which can be user-defined) before it declares that this Helper is gone (or dead) and initiates the packet rescheduling procedure (to be discussed in the next section).

### Packet Scheduler

It has been shown that MPTCP, which schedules packets based on RTT, may suffer from the biased-feeding problem [10], in which, most of the time, only the fastest link is selected to send data, while all the other links are not utilized. In addition, in CBSP, given that different Helpers might connect to the Internet through different ISPs, their path characteristics (e.g., delay and bandwidth) might be significantly different, which could lead to excessive out-of-order packets and queuing delay at the CBSP server (for uplink traffic) and the requestor (for the downlink traffic).

We thus propose a Scheduled Window-based Transmission Control (SWTC) to reduce the queuing delay at the CBSP server and the requestor. The basic idea here is to schedule each packet to be sent out through the “fastest” Helper when the requestor or the CBSP server has a packet to send. To estimate the fastest Helper, we calculate the expected duration required from transmitting a data segment until receiving its corresponding ACK. For the sake of simplicity, we assume that the link characteristics between each Helper and the requestor are either similar or insignificant compared to the variations of path characteristics between different Helpers and the CBSP server. Therefore, when scheduling packets, we only consider the path conditions between the CBSP server and each Helper. The fastest Helper is defined as the one with the minimum expected delay (denoted by *D*_*i*_ for path *i*). The delay *D*_*i*_ consists of two parts: the waiting time *w*_*i*_ from now until the packet can be injected into a particular link and the round-trip network delay *r*_*i*_.

Given a packet loss probability *p*, based on a prior study [31], we can estimate the expected value of delay *E*(*D*_*i*_) as

$$E_{i}(w_{i},r_{i},p_{i},q_{i})=r_{i}\times \left(\frac{1}{{\left(1-p_{i}\right)}^{2}}-\frac{1}{\left(1-p_{i}\right)}+1\right)+w_{i}+q_{i},\;\forall Path\;i$$ (2)

where *r*_*i*_ is the network round-trip delay (i.e., the duration between when the scheduled packet is injected into the link and when the requestor receives the corresponding ACK), *p*_*i*_ is the packet loss probability, *w*_*i*_ is the waiting time before the scheduled packet can be injected into the link, and *q*_*i*_ is the estimated queuing delay [19] on path *i*.

**Fig 7:**
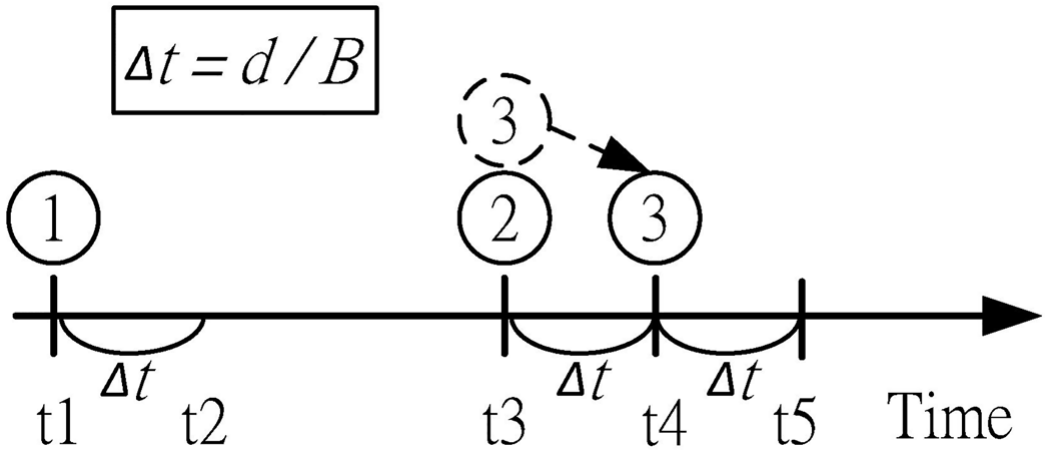
Example of estimating *m*_*i*_.

Two things need to be considered to estimate *w*_*i*_: the time *m*_*i*_ to wait for all previous scheduled packets to be injected into the link and the time *n*_*i*_ to wait until the congestion window is open (i.e., when cwnd is larger than the amount of outstanding data). That is, *w*_*i*_ = *m*_*i*_ + *n*_*i*,_ ∀*Path i*, *m*_*i*_ can be computed by estimating the transmission delay of all previous scheduled packets. The transmission delay can be obtained by dividing the available bandwidth (*B*_*i*_) by the packet size (*d*_*i*_). We adopt the bandwidth estimation function of TCP Westwood [32] to calculate *B*_*i*_ and assume that all the packets have the same size. In the example shown in Fig 7, packet 1 is sent at *t1*, and the Sink receives its ACK at *t3*. The ACK of packet 1 triggers the scheduled transmission of packets 2 and 3. In this case, the waiting time *m*_*i*_ for packet 2 is zero. Alternately, given that packet 3 needs to wait for packet 2 to be transmitted, the estimated *m*_*i*_ for packet 3 will be *Δt*.

**Fig 8:**
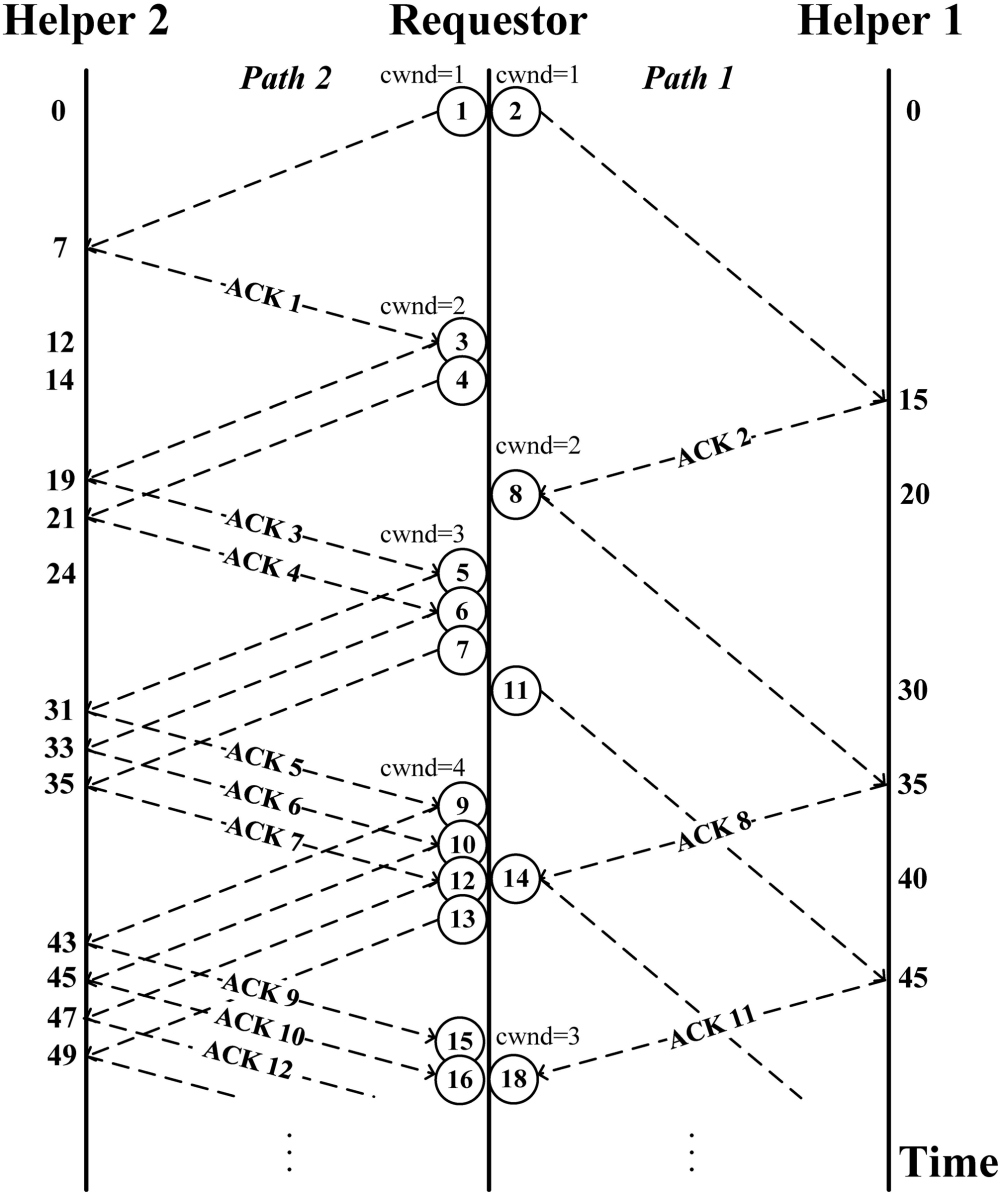
Example of scheduled window-based transmission control. Transmission Delay: Path 1 = 10, Path 2 = 2; Propagation Delay: Both paths are 5.

*n*_*i*_ is the estimated time to wait until there is enough bandwidth to transmit the packet (i.e., cwnd > the amount of outstanding data). In other words, *n*_i_ estimates the time needed for enough ACKs to come back so that cwnd can be advanced to a level that is larger than the amount of outstanding data on path *i*. Finally, we use the measured RTT to estimate the network round-trip delay *r*_*i*_ for path *i*. An example of the SWTC operation is shown in Fig 8 (we assume that the loss rate *p* is zero for both paths and that the round-trip delays are *r*_*1*_ = 20 and *r*_*2*_ = 12); when the sender receives ACK 1 from path 2 at time 12, the *n*_*2*_ for path 2 is zero. Alternately, given that the arrival time for the earliest ACK to come back from path 1 is at time 20, the *n*_1_ for path 1 is 8 (= 20–12). To estimate *n*_i_, when a data segment *k* is sent out through path *i* at time *t*, the requestor records the estimated ACK segment arrival time (*est_ACK_AT*_k_) for the data segment *k*. Specifically, *est_ACK_AT*_k_ = *t* + *smoothed rtt*_*i*_ + *m*_*i*_ of path *i*, where *smoothed rtt* is the average measured rtt of all the received data segments [33].

We can predict when the ACK of data segment *k* will arrive according to *est_ACK_AT*_k_. As shown in Fig 8, assuming the *smoothed rtt* of path 2 is 12 at the time ACK 1 is received, the *est_ACK_AT* of data segment 3 will be 24 (12 + 12 + 0). In other words, we expect that the ACK of data segment 3 will arrive at time 24. Alternately, the *est_ACK_AT* of data segment 4 will be 26 (12 + 12 + 2), assuming that the transmission delay for sending out data segment 3 is 2. The requestor also needs to calculate the *est_ACK_AT* for each scheduled data segment. This is because it is possible that the cwnd might still not be large enough, even when all the ACKs of the outstanding data have been sent back. Note that the use of SWTC does not exclude the need to have a reordering buffer because, in practice, perfect scheduling is difficult to achieve in a very dynamic network environment. Furthermore, in addition to the use of multiple paths, packet reordering can be due to other causes, such as route fluttering and router forwarding lulls [34].

If the helper manager component of the requestor detects that a Helper has left the network, it signals the packet schedulers in the requestor and the CBSP server (through the fastest path) for packet rescheduling. The packet scheduler will move all of the outstanding packets in the retransmission queue (RTQ) and the scheduled queues (SQ) for each Helper to the rescheduling queue (RSQ) (at that point, RTQ stores the packets already sent but not yet ACKed, while the SQs have the packets scheduled but not yet sent to the Helpers.) These MPTCP packets in RSQ are sorted based on their DSN. The packet scheduler then takes packets in RSQ as the source and runs SWTC again on the remaining Helpers. Note that the packets in the transmission queue (TxQ) and the Helper selection process will be held until all the packets in RSQ have been processed by SWTC. In other words, no packets in TxQ will be scheduled and no new Helpers can join the network until all the packets in RSQ have been scheduled. Finally, a similar rescheduling procedure will be triggered when a new Helper joins the network.

### Accounting Management

To use the CBSP service, all CBSP clients need to first register themselves with the CBSP server and setup their accounts. To create accounting information for the requestors and the Helpers, the CBSP server continuously monitors all the traffic it forwards and identifies the requestors and the Helpers through the headers of the tunneled IP-in-IP packets (for uplink traffic) and NAT port numbers (for downlink traffic). Based on this recorded accounting information at the CBSP server, the requestors and the Helpers can then be charged and awarded, respectively, via various virtual credit mechanisms, such as PayPal [35].

## Performance Evaluation

**Fig 9:**
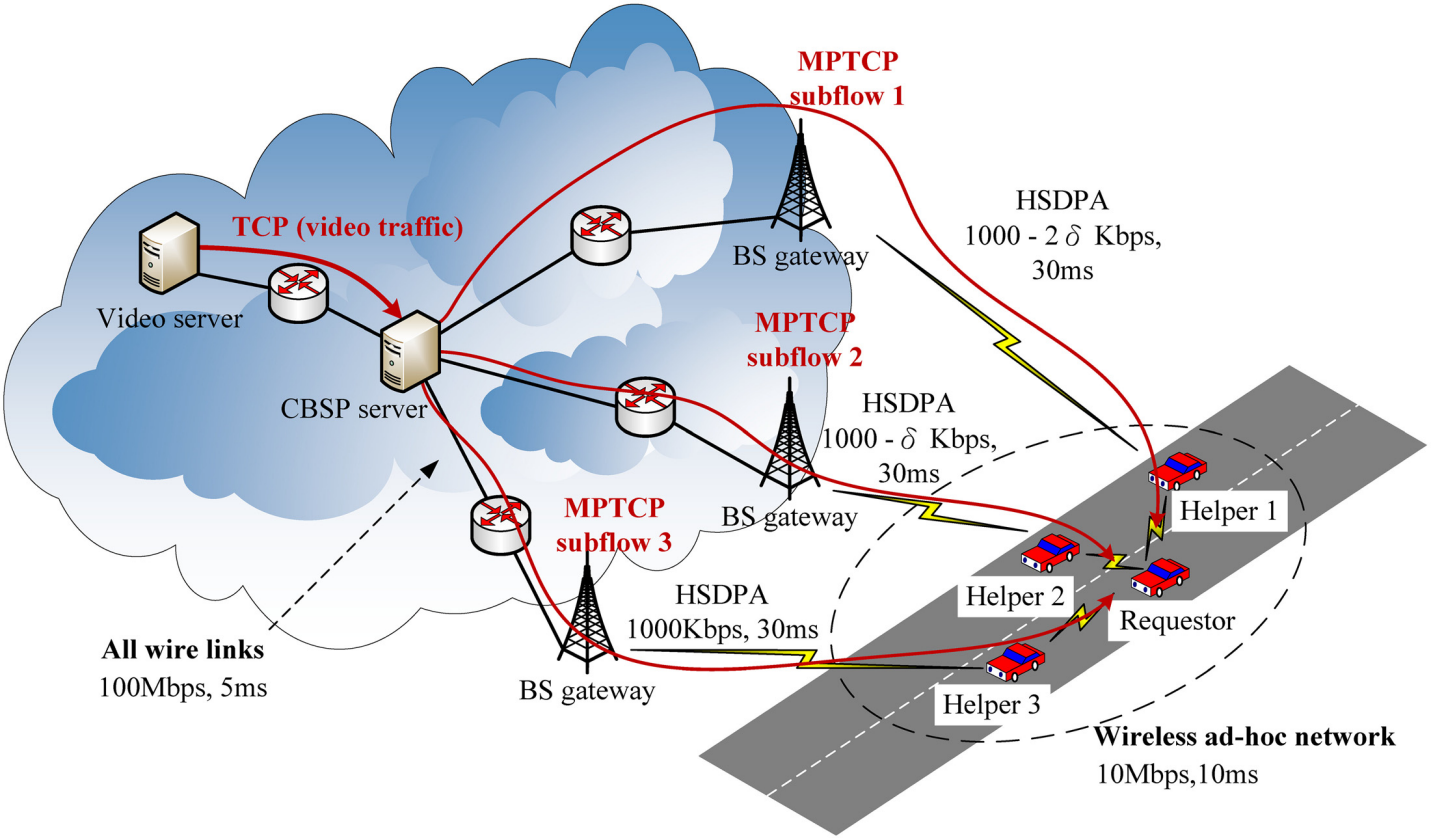
The simulated topology.

In this section, we evaluate the performance of CBSP for different Helper selection strategies and packet scheduling algorithms. We consider a scenario in which a requestor is downloading videos from a video server through the help of three neighboring nodes using CBSP. Real-world YouTube data, which has an ON-OFF pattern that is associated with the ‘initial burst’ and ‘throttling’ phases of the YouTube protocol [36], is used to simulate the video traffic (for the ease of discussion, in this paper we do not consider YouTube traffic that is based on newer adaptive protocols (e.g. DASH [37]) and leave that as our future work). We assume the bandwidths of these three Helpers differ by *δ*. Fig 9 shows the simulated topology, where the requestor is connected to three Helpers whose bandwidths are 700, 700 − *δ* and 700 − 2*δ* kbps, respectively. We let the propagation delay for all the Helpers be the same (assuming that they have similar distances to the cellular tower). For simplicity, we also assume that the forwarding delay of each Helper is the same. We compare CBSP with Co-MPTCP [8], which has a similar architecture to CBSP but adopts RTT-based scheduling (while CBSP uses SWTC scheduling). Both protocols are implemented in ns-2 [38]. The performance metrics used in this section are PCI (playout continuity index) and startup delay. Due to the space limitation, in this paper we do not discuss transport layer metrics like throughput or goodput since they are less important as compared to PCI in the context of video playout.

PCI measures the continuity of video playback in an end device [16] and is defined as *PCI* = 1 –*pause time/playout time*, where pause time is the total pause duration experienced by the viewer and playout time is the total duration of successful video playback. A higher PCI value indicates better video quality and user perception. The startup delay is the time taken, at the beginning of the video transmission, to buffer a portion of the video before the viewer can start watching the video. Most smartphones implement a playout buffer to buffer video packets before playing them on the device. For example, all Android device uses a LowWaterThreshold to decide when the download should resume during video playout [39]. In our simulations, we set the LowWaterThreshold as 316 KB (i.e. 2 video chunks or segments). We evaluate the performance of CBSP in a lossy environment with different types of loss distributions.

We first compare the Helper selection strategy used by CBSP (as discussed in Section 4) with other approaches, such as high-bandwidth-first [40] and long-encounter-first [28]. We use the trace from an earlier work [41] to simulate the mobility of the Helpers and assume that the requestor needs a minimum bandwidth of at least 1600 kbps and that the bandwidth of the Helper nodes ranges between 100 and 1000 kbps. If the aggregate bandwidth of the neighboring Helpers is less than the required minimum bandwidth, the requestor continues broadcasting INVITE to find more Helpers. Here, we consider two cases. One is when the bandwidth variation between Helpers (i.e., *δ*) is small, and the other is when *δ* is large.

**Fig 10:**
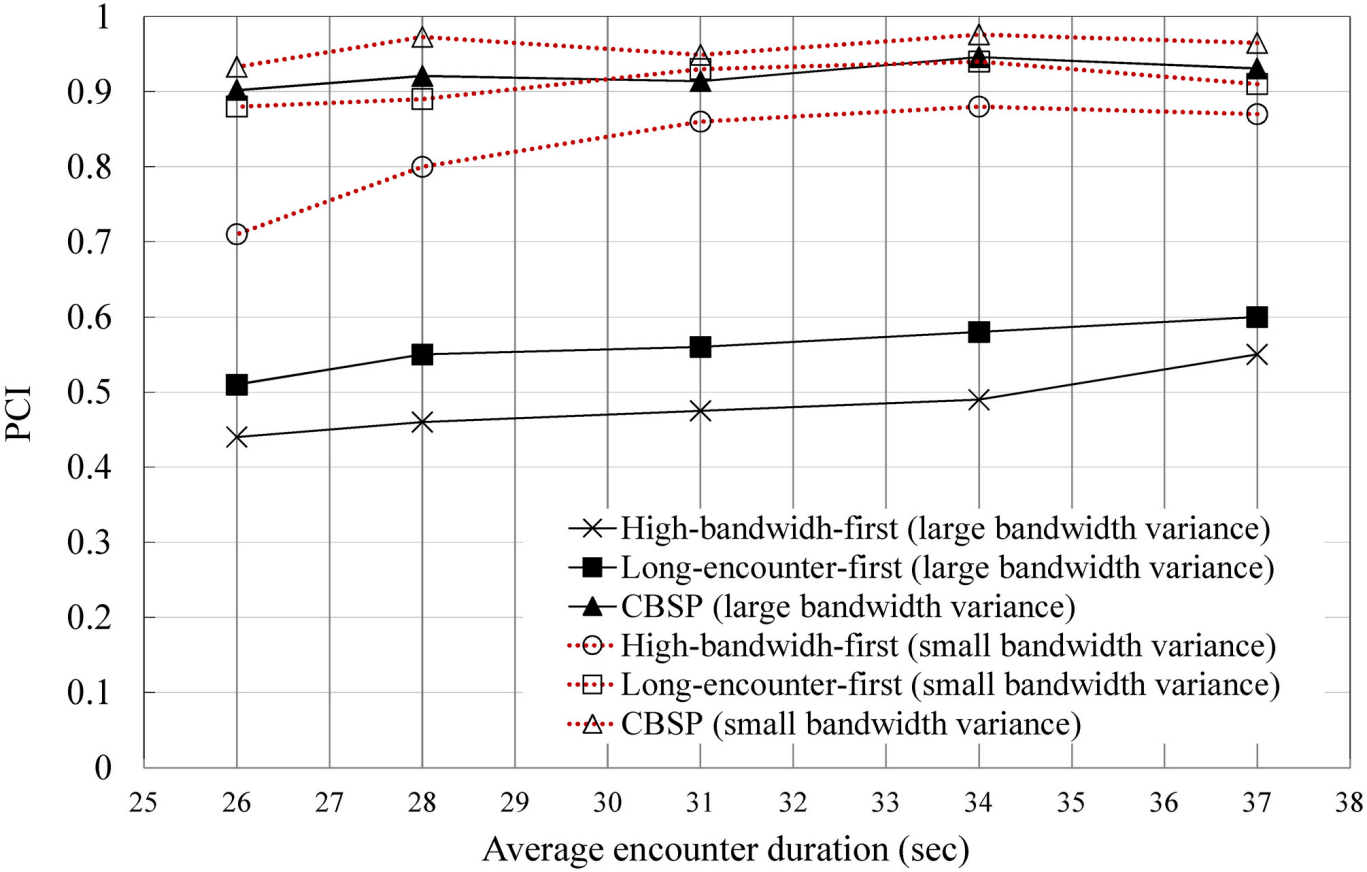
The effect of different Helper selection strategies.

The results show that CBSP generally has a better PCI than the other two Helper selection strategies, as shown in Fig 10. The small *δ* case tends to have a better PCI than the large *δ* case because the former usually has a smaller reordering delay than the latter. In addition, the disjoin of a Helper in the large *δ* variance case often has a more harmful impact on the network performance than the other case, especially when the high-bandwidth-first strategy is used for selecting the Helpers. Unlike the other two Helper-selection strategies, in CBSP, the PCIs of these two cases are not significantly different because CBSP will be less affected by the bandwidth heterogeneity and tend to select a similar set of candidate Helpers. Finally, long-encounter-first performs slightly better than high-bandwidth-first in both cases because it can reduce the chance of disjoining the Helpers and thus rescheduling (although at the cost of a larger reordering delay at the CBSP server). In our experiments, we find that the disjoining of Helpers generally has a more adverse effect on the PCI than the increased queuing at the CBSP server.

**Fig 11:**
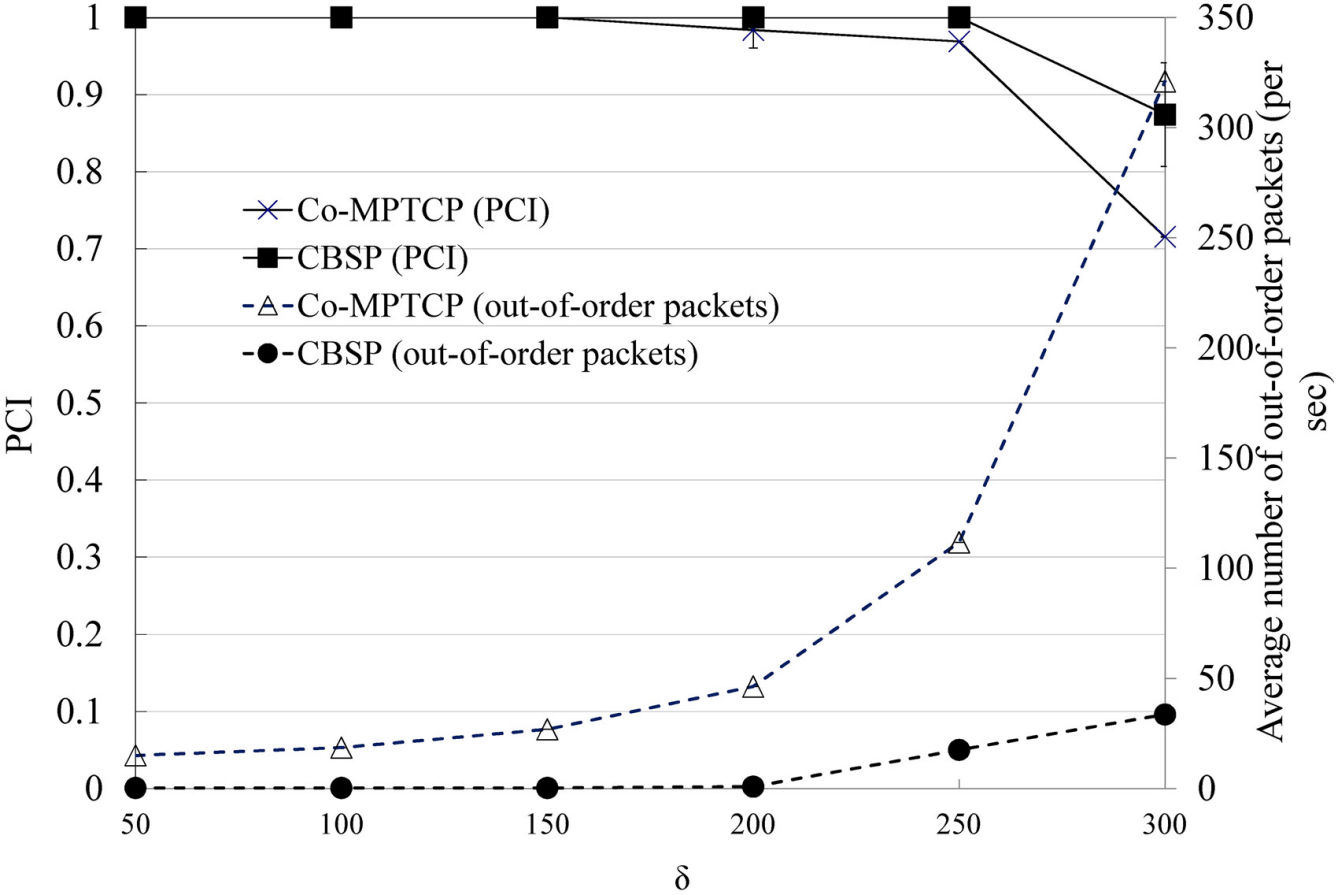
The PCI and the number of out-of-order packets when varying *δ*.

**Fig 12:**
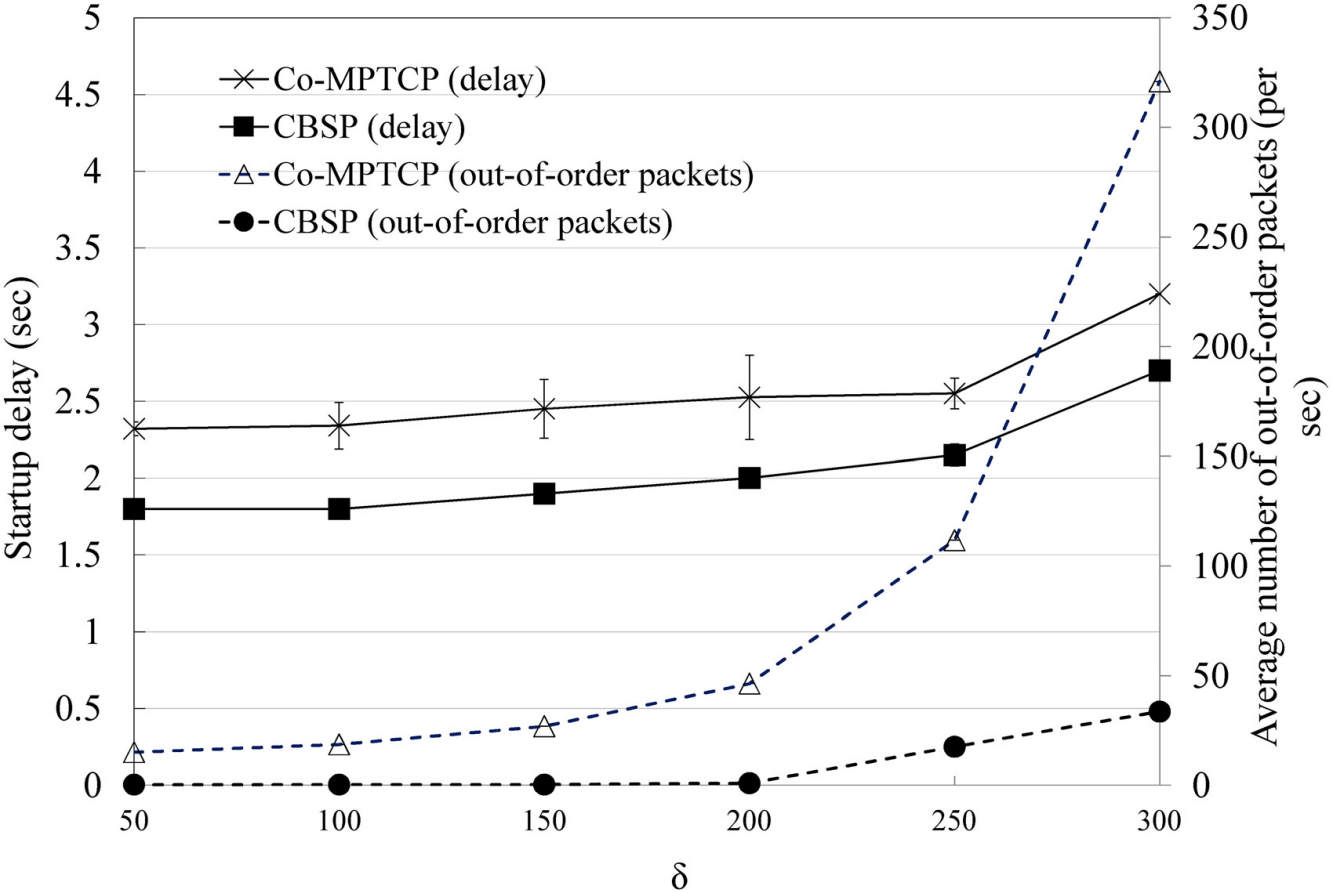
The startup delay and number out-of-order packets when varying *δ*.

We next consider the performance of packet scheduling in CBSP (i.e., SWTC) based on the topology shown in Fig 9 and compare it with Co-MPTCP (which implements RTT-based scheduling, as in MPTCP). In this experiment, we assume that the requestor tries to download a video from the server (with a bit rate of 1271 kbps) with the assistance of three static Helper nodes, the bandwidths of which are 700, 700 − *δ*, and 700 − 2*δ* kbps, respectively. As shown in Figs 11 and 12, CBSP generally performs better than Co-MPTCP in terms of PCI and startup delay. When *δ* is small (e.g., less than 200), although Co-MPTCP tends to have more out-of-order packets than CBSP, they have similar PCIs because of the use of a playout buffer at the requestor. The startup delay is generally a function of the configured LowWaterThreshold, the available bandwidth, and the number of out-of-order packets, as shown in Fig 12.

**Fig 13:**
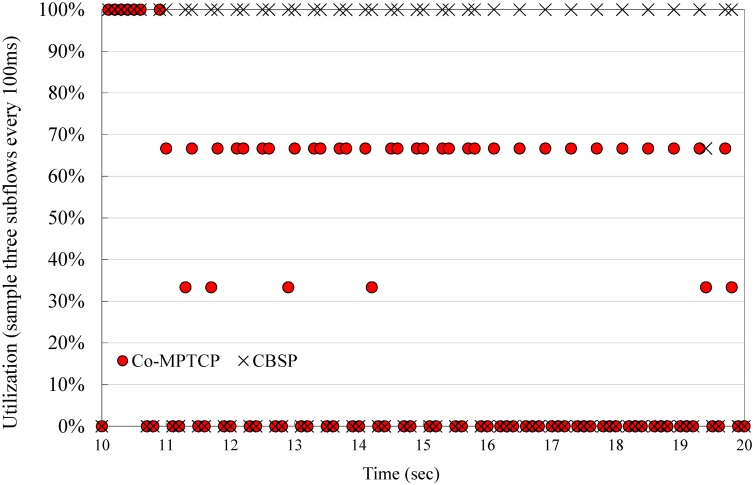
Utilization of subflows/Helpers when using CBSP vs. Co-MPTCP.

Fig 13 shows the utilization of Helpers/subflows when different scheduling methods are employed in CBSP and Co-MPTCP for a small *δ* (*δ* = 50). We examine each subflow every 100 ms to see if it is idle (i.e., no packet is scheduled on that subflow) and define *utilization* as the “*number of active subflows/total subflows*.” Taking Fig 9 (with a total of three subflows) as an example, 100% utilization means that all three subflows are active, and 66% utilization suggests that one of the subflows is idle. CBSP generally achieves a better utilization of the Helper nodes than Co-MPTCP because it utilizes both network characteristics (e.g., RTT, available BW, and loss) and TCP protocol behavior (e.g., cwnd) to estimate the link delay associated with each Helper. Those periods with zero utilization shown in Fig 13 mostly happen during the OFF period of the video traffic [36].

**Fig 14:**
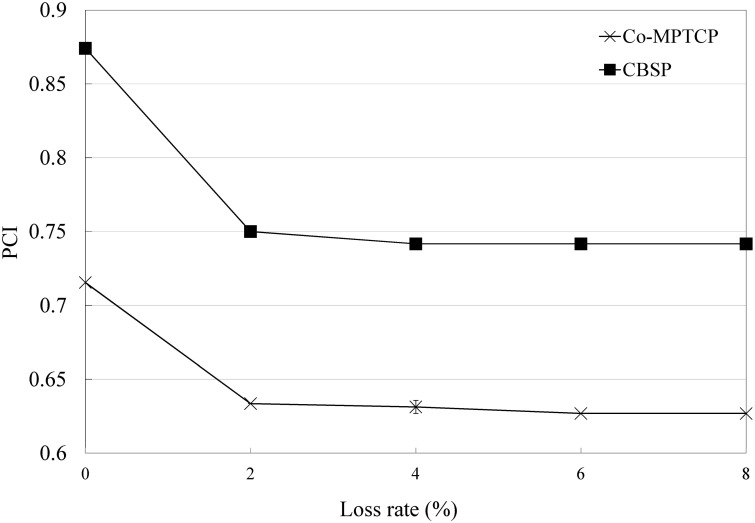
Comparison of CBSP and Co-MPTCP in a lossy network (PCI).

**Fig 15:**
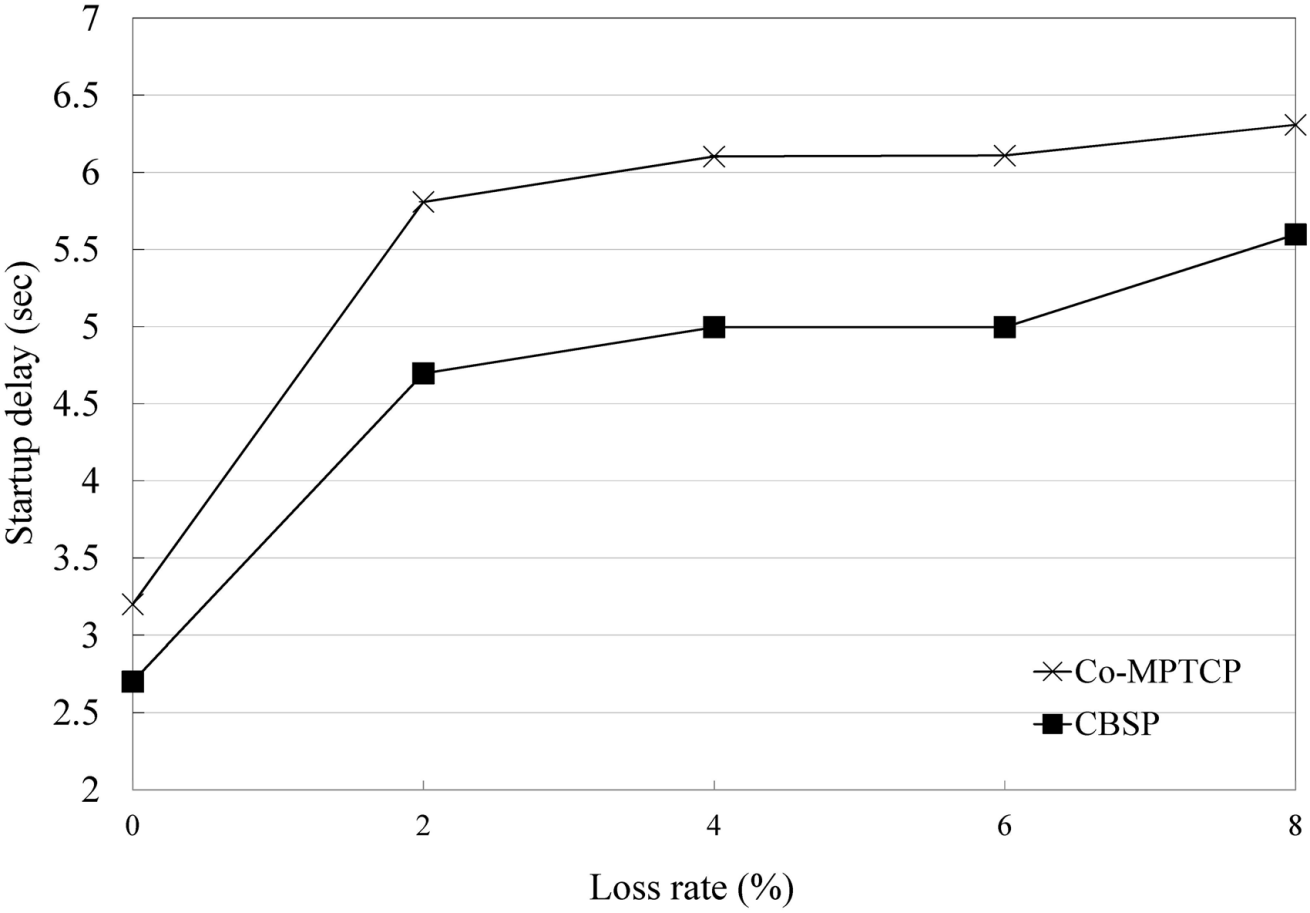
Comparison of CBSP and Co-MPTCP in a lossy network (startup delay).

In the above experiments, we assume that there is no packet loss during the video downloads. Next, we discuss the performance of CBSP when some packets are lost. For simplicity, in this experiment, we model subflow 3 in Fig 9 as a lossy link, and the packet loss follows a uniform distribution. We find that, as shown in Figs 14 and 15, CBSP generally performs better than Co-MPTCP under different loss rates because it considers the loss rate in its scheduling.

## Discussion

In this section, we briefly discuss how the default TCP receiver window size on the smartphone, the video bitrate, and the video protocol will affect the performance of CBSP.

**Fig 16:**
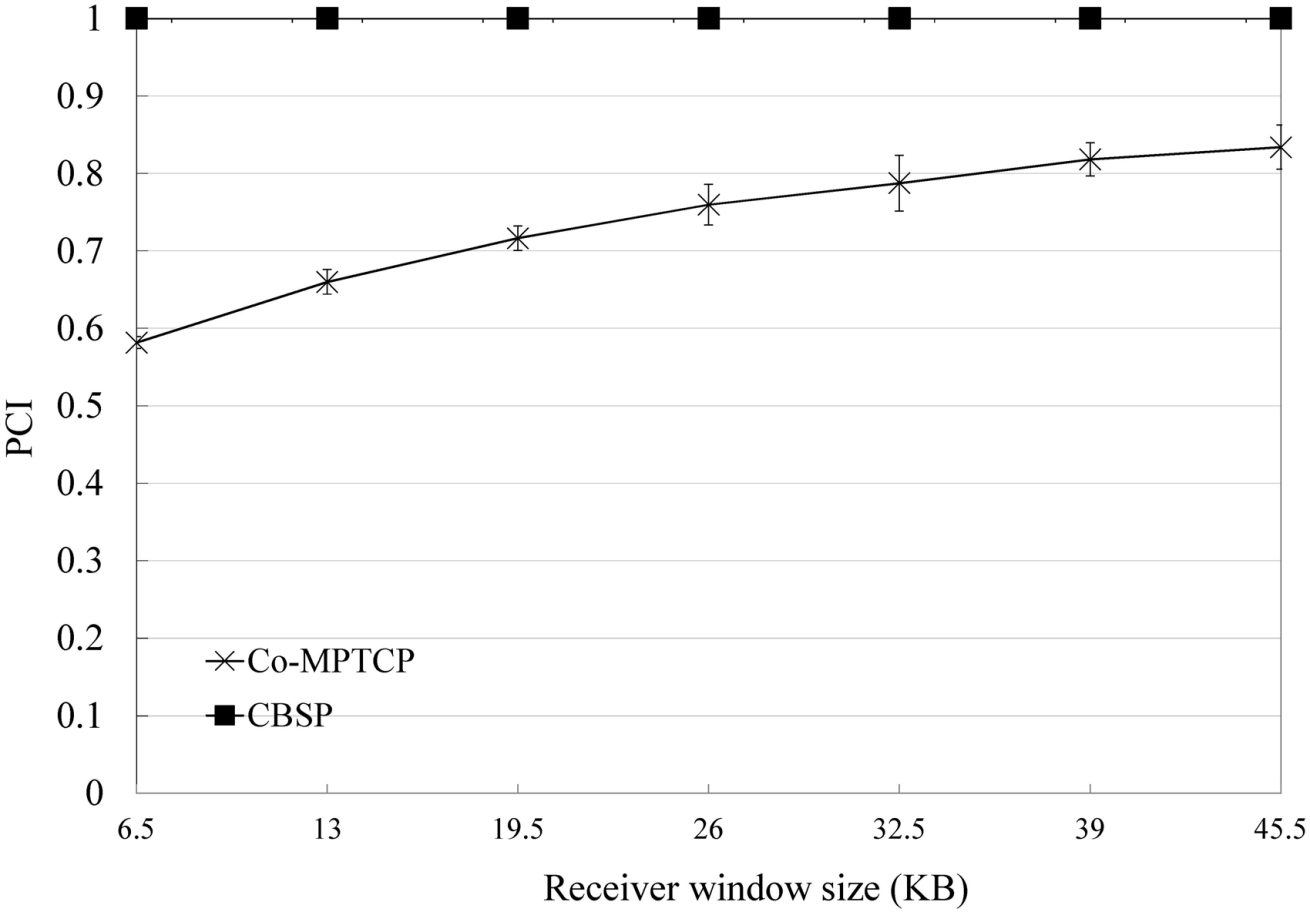
Performance of CBSP for various default receiver window sizes in PCI.

**Fig 17:**
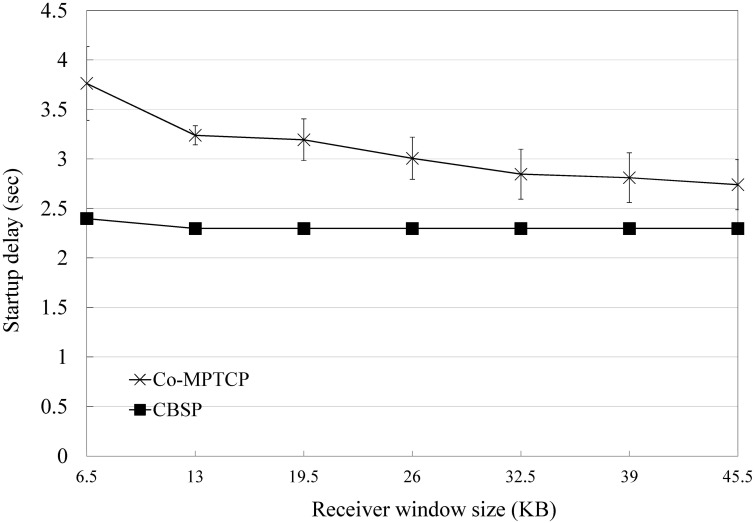
Performance of CBSP for various default receiver window sizes in startup delay.

Previous studies have shown that when using a multipath transfer protocol (such as MPTCP), a large number of out-of-order packets could introduce a problem called Window-Induced Receiver Buffer Blocking [42], especially when the receiver buffer is limited (the default receiver window size used by an Android phone typically ranges from 8K to 500K). The receiver window is calculated as the sum of the Advertised Receiver Window (A_RWND) and the amount of outstanding data (i.e., the out-of-order packets) [42]. A TCP sender will reset its congestion window (cwnd) to A_RWND when it finds its current cwnd is larger than A_RWND. Compared to Co-MPTCP, CBSP generally has less of out-of-order packets and hence is less affected by the default receiver window size, as shown in Figs 16 and 17.

**Fig 18:**
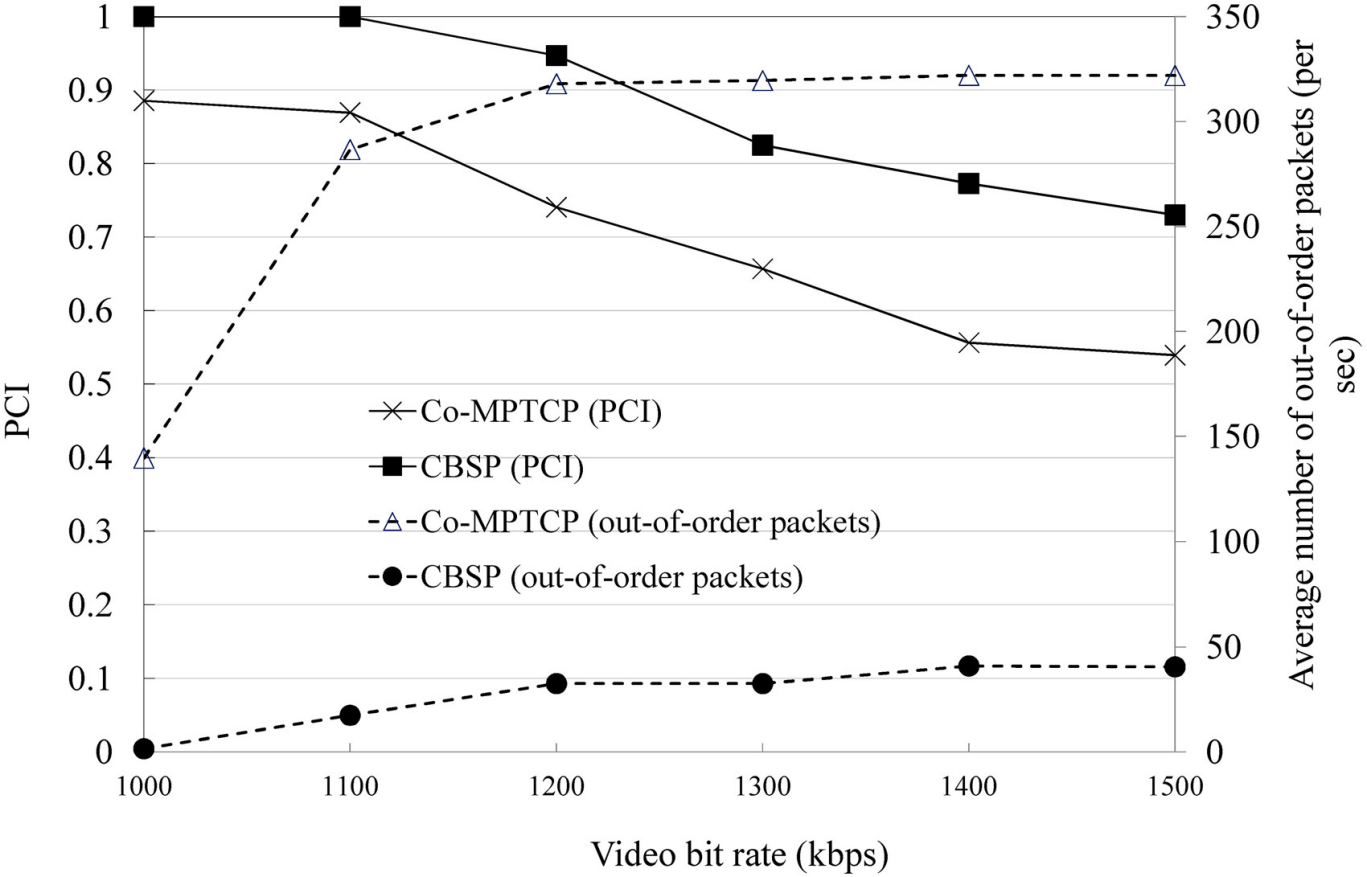
Comparison of PCI with varying traffic data rates.

In YouTube, the video sending rate from the server is usually slightly larger than the playout rate (more specifically, sending rate = playout rate * throttle-factor, where here the default of *throttle-factor* is 1.25). When viewing high-resolution videos, the server sending rate could possibly be higher than the aggregated bandwidth of multiple links and thus saturate all the links. As shown in Fig 18 (*δ* = 300), the number of out-of-order packets remains the same when the links are saturated, and PCI becomes worse as the video bit rate increases.

**Fig 19:**
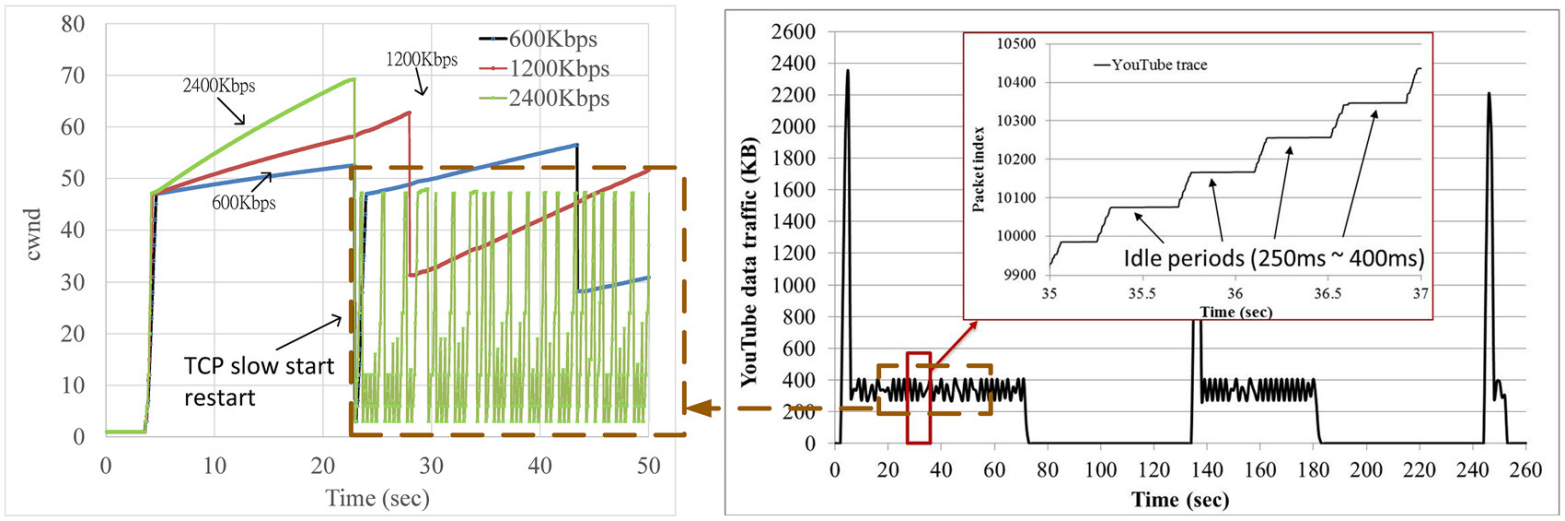
An example showing frequent slow start restart events observed on the faster link.

YouTube traffic typically has an on-off pattern [36]. We used a packet sniffer (Wireshark [43]) on the smartphone to record real Youtube data (we did not record any video content but only header information such as packet length, packet arrival time, and so on, which complies to Youtube's Terms of Service. All the packet traces we collected from Youtube can be found at https://www.youtube.com/watch?v=xpT7av-kiXg.). Based on our observations of these data, its off period typically ranges between 250 ms and 400 ms in the throttling phase. According to RFC 5681 [44], TCP slow start will be triggered if a TCP connection has not sent data in an interval larger than its RTO. We performed a trace-driven simulation and observed that many slow start events (and hence reduced cwnd) occur on the fastest link, as shown in Fig 19. Previous studies have shown that the average RTT observed in 3G networks is approximately 98 ms [45] (and approximately 60 ms in 4G networks [46]), which suggests that such cases can also happen in the real world.

**Fig 20:**
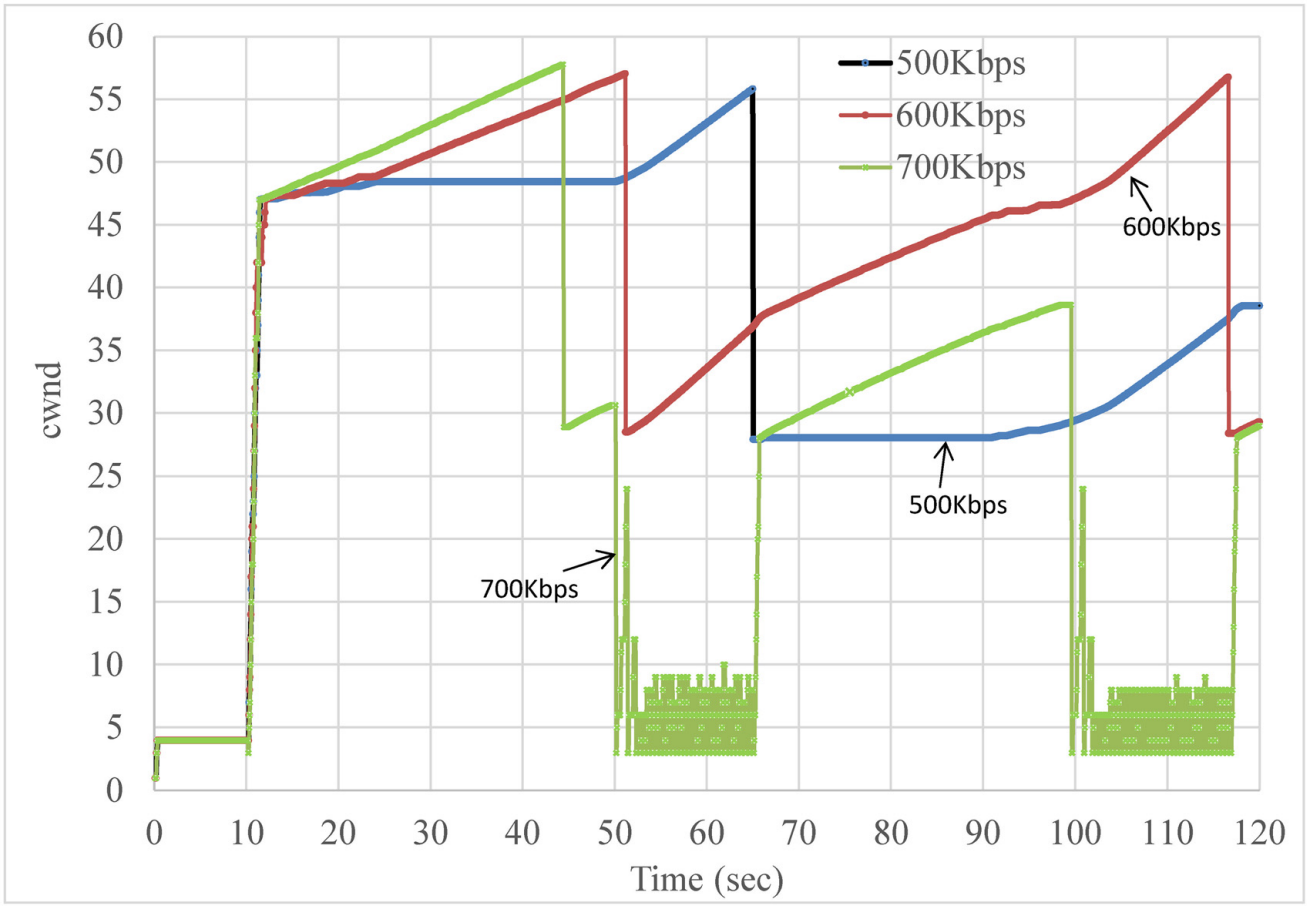
A snapshot of cwnd in an OFF period = 380 ms case in Co-MPTCP.

**Fig 21:**
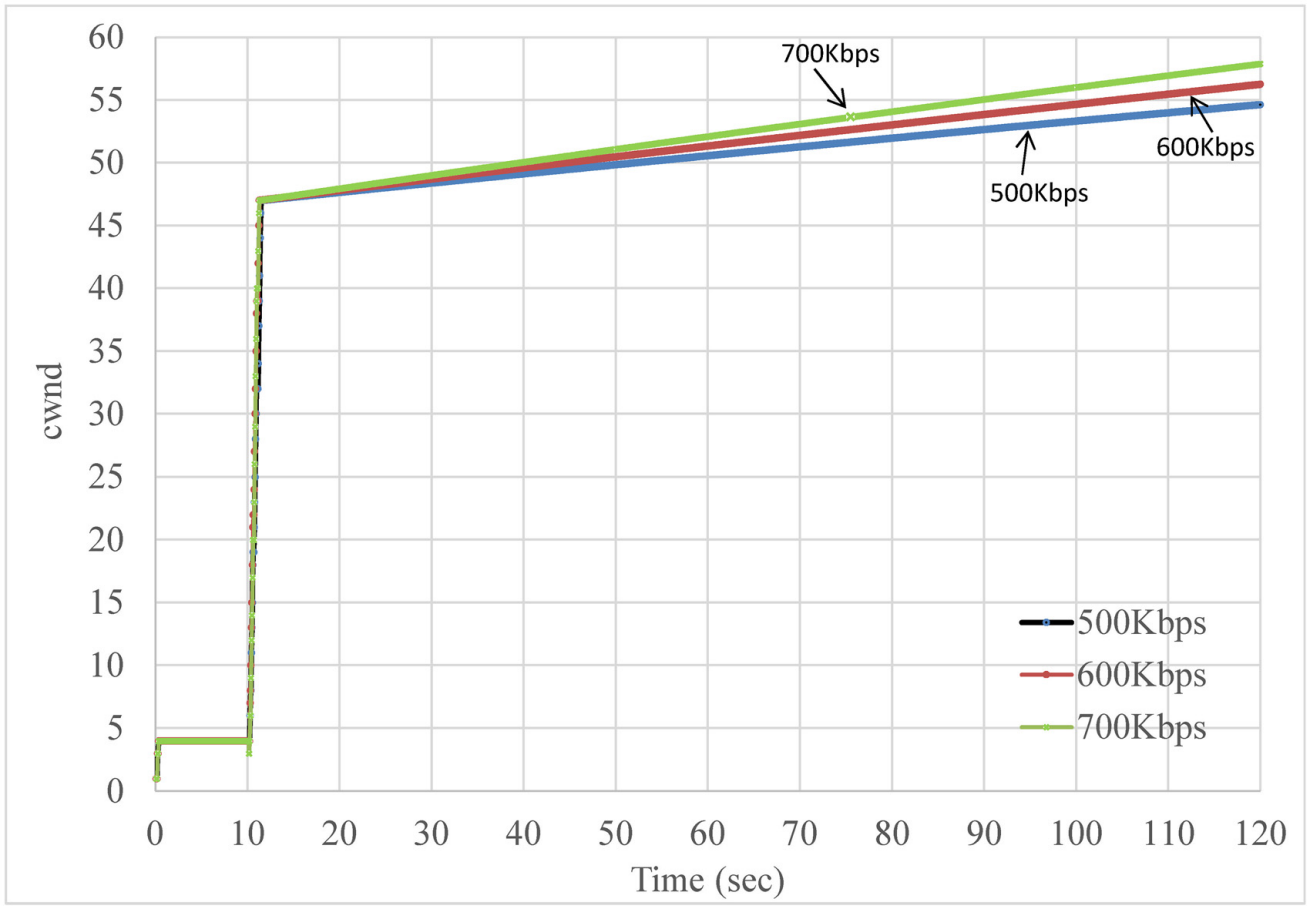
A snapshot of cwnd in an OFF period = 380 ms case in CBSP.

As shown in Figs 20 and 21, CBSP does not trigger TCP slow starts as frequently as Co-MPTCP, as the pre-scheduling scheme of CBSP allows it to ‘pace’ the packets more evenly.

**Fig 22:**
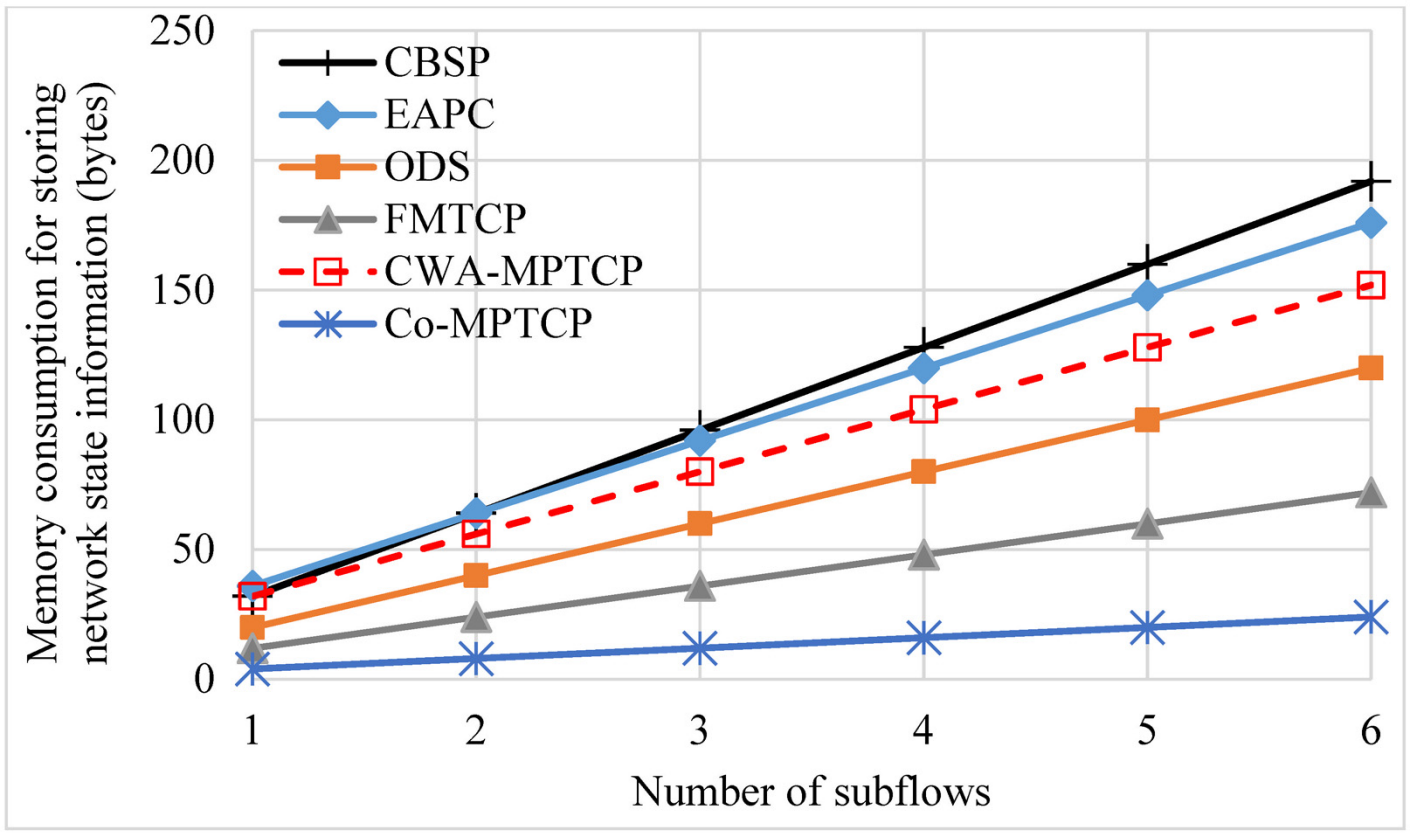
Comparison of CBSP with other protocols in memory consumption.

Note that, while our simulation results show that CBSP performs better than Co-MPTCP [8], it could incur extra overhead in terms of memory consumption (for storing extra network state information) and computation overhead. We perform some analysis and simulation experiments to evaluate these overhead, as shown in Fig 22 and Table 2. Here *n* referring to the number of the flows, and we assume that it takes 4 bytes to store each network state information. Our results suggest that the extra overhead required by CBSP might be acceptable compared to other existing protocols.

**Table 2:** Comparison of time complexity of the algorithm.

Protocols	Complexity
**CBSP**	*O*(*n*)
**EAPC** [10]	*O*(*n*)
**ODS** [19]	*O*(*n*)
**FMTCP** [21]	*O*(*n*)
**CWA-MPTCP** [20]	*O*(*n*)
**Co-MPTCP** [8]	*O*(1)

Finally, due to the limitation of the scope and space of this paper, in this work we assume that the helpers are trustworthy and do not consider possible security attacks to the phones. Several prior studies [47–50] have proposed various mechanisms to build security capability into mobile devices. We will investigate this matter in our future work.

## Conclusion and Future Work

In this paper, we propose a collaborative bandwidth sharing protocol (CBSP) built on top of MultiPath TCP (MPTCP). CBSP enables users to buy bandwidth on demand from neighbors (called Helpers) and utilize these as virtual interfaces to bind the subflows of MPTCP to avoid modifying the implementation of MPTCP. In addition, we design an algorithm called Scheduled Window-based Transmission Control (SWTC) to tackle the biased-feeding problem introduced by the RTT-based scheduling originally used by MPTCP. We perform extensive simulations to evaluate the performance of SWTC. We are currently implementing CBSP on a range of smartphones to evaluate its performance in the real world.

As for future work, we plan to extend CBSP in following directions. (1) We will try to find a guaranteed delay bound for packet reordering to improve the performance of SWTC. (2) Due to space limitations, we do not discuss the pricing scheme used in CBSP in this work. We will thus examine this issue in a separate paper using game theory, with the goal of optimizing both network utilization and the Helpers’ profit. (3) Finally, security management and how to incorporate UDP traffic into the current framework are also important open research issues we plan to study in the future.
