# Correction: Avoiding Biased-Feeding in the Scheduling of Collaborative Multipath TCP

**DOI:** 10.1371/journal.pone.0167183

**Published:** 2016-11-18

**Authors:** Meng-Hsun Tsai, Chien-Ming Chou, Kun-chan Lan

There are errors in the Funding section. The correct funding information is as follows: This research was partially supported by the Ministry of Science and Technology, Taiwan, R.O.C., under the grant numbers: MOST 104-3115-E-009-006-, MOST 104-2221-E- 006-041- and MOST 104-2221-E-006-024-, https://www.most.gov.tw. The funders had no role in study design, data collection and analysis, decision to publish, or preparation of the manuscript. There was no specific funding for the rest of this project. There was no additional external funding received for this study.
